# TNF-**α** blockade mitigates immune checkpoint–related nephritis in a humanized mouse model

**DOI:** 10.1172/jci.insight.199694

**Published:** 2026-03-05

**Authors:** Victor D. Cuenca Narvaez, Coraima Nava Chavez, Omar Al Refai, Johanna E.J. Jacobs, Luis E. Gutierrez, Song Zhang, Xiaoyan Li, Jacob B. Hirdler, Michael F. Romero, Joerg Herrmann, Xiaogang Li, Haidong Dong, Alfonso Eirin, Sandra M. Herrmann

**Affiliations:** 1 Department of Internal Medicine, Division of Nephrology and Hypertension,; 2 Department of Cardiovascular Medicine,; 3 Department of Biochemistry and Molecular Biology,; 4 Department of Immunology,; 5 Department of Physiology and Biomedical Engineering, and; 6 Department of Urology, Mayo Clinic College of Medicine and Science, Rochester, Minnesota, USA.

**Keywords:** Immunology, Inflammation, Nephrology, Cancer immunotherapy, Cellular immune response, Mouse models

## Abstract

Immune checkpoint inhibitors (ICIs) can cause immune-related adverse events (irAEs), with acute interstitial nephritis (ICI-AIN) being the most common irAE. While the exact mechanism remains unclear, upregulation of IFN-γ and TNF-α pathways has been implicated. This study used a humanized chimeric PD-1/PD-L1 mouse model to assess renal effects of ICIs, alone or combined with proinflammatory cytokines, and to test if selective TNF-α blockade could prevent ICI-AIN. Mice were randomly divided into 4 experimental groups: Control, ICI-Only, ICI-Cytokines (ICI-Cyt), and ICI-Block (ICI-TNF-α blockade). Renal function and cytokine profiles were assessed, while kidney tissue was analyzed using microscopy and single-cell RNA-seq. Histology revealed increased renal infiltration of CD4^+^/CD8^+^ T cells in ICI-treated groups and decreased TNF-α expression following TNF-α blockade. Additionally, kidney tissue ELISA demonstrated reduced IFN-γ levels following TNF-α blockade. Plasma IL-6, MCP-1, and TNF-α were lower in ICI-Block mice. Single-cell RNA-seq revealed shifts in immune cell populations and genes of interest including *Bcl2a1*, *Icos*, *Il18r1*, *Ccr2*, and *Jaml*. This humanized model replicates ICI-AIN key features, revealing a synergistic role of ICIs and proinflammatory cytokines. TNF-α blockade demonstrated protective effects, supporting its potential role in mitigating the risk of ICI-AIN.

## Abstract 2

<style type=”text/css”>a { text-decoration: none; color: #464feb; } tr th, tr td { border: 1px solid #e6e6e6; } tr th { background-color: #f5f5f5; } </style> A humanized mouse model shows that TNF‑α blockade reduces kidney inflammation caused by immune checkpoint inhibitors, suggesting a potential strategy to prevent immune‑related kidney injury.

## Introduction

Immune checkpoint inhibitors (ICI) are a class of anticancer immunotherapies that have significantly improved survival in patients with a wide range of malignancies (1, 2).

These ICIs are monoclonal antibodies that act by blocking immune checkpoints (e.g., programmed cell death protein 1 [PD-1], programmed cell death ligand 1 [PD-L1], and the cytotoxic T lymphocyte antigen 4 [CTLA-4] signaling pathway) (1, 3, 4). This blockade leads to a break of immune tolerance by unleashing quiescent tissue-specific self-reactive T cells, potentially leading to development of immune-related adverse events (irAEs) (1, 5). Acute interstitial nephritis (AIN) is the most common cause of acute kidney injury (AKI) in patients treated for ICI accounting for approximately 90% of cases in the largest published multicenter cohort, with significant implications increasing morbidity and mortality (3, 6–9).

The exact mechanism of ICI-induced AIN (ICI-AIN) remains unclear. Previous studies have shown upregulation of INF-γ and TNF superfamily signatures in kidney biopsies from patients with ICI-AIN compared with non-AIN AKI controls (10–12). The optimal management of ICI-AIN is also uncertain. TNF-α blockade has demonstrated effectiveness in patients with steroid-dependent or refractory ICI-AIN, and as salvage therapy in cases with partial or no kidney recovery, without negatively affecting disease outcomes (7, 13–15). Preventing the development of AKI may decrease healthcare costs and improve survival. However, prevention efforts have been hampered by a lack of understanding of the mechanisms underlying ICI-AIN (5, 16).

In this study, we used a humanized chimeric mouse model to investigate the mechanisms of ICI-AIN and evaluate preventive strategies. We aimed to induce kidney injury through ICI therapy, assess the effect of costimulation with TNF-α and IFN-γ, and examine the therapeutic potential of TNF-α pathway blockade.

## Results

### Systemic characteristics

Demographic characteristics within experimental groups are presented in Table 1. Nephritis status was determined according to histopathological criteria described in detail in the Supplemental Methods (supplemental material available online with this article; https://doi.org/10.1172/jci.insight.199694DS1). In that context, in the ICI-Only group, 66.7% of mice developed nephritis, with a mean inflammation score of 1, consistent with mild nephritis. In contrast, in the ICI-Cyt group, 100% of mice developed a degree of nephritis, with mean inflammation score of 2.1, indicating mild to moderate severity overall. Detailed demographic characteristics by nephritis status and histological evaluation are provided in Supplemental Table 1 and Supplemental Methods.

### Kidney function parameters

Mice in the ICI-Only group exhibited higher plasma creatinine and a trend toward increased BUN compared with controls (*P* = 0.08; Supplemental Figure 1, A and B). The ICI-Cyt group showed significantly higher BUN levels versus controls (Supplemental Figure 1B). When stratified by nephritis status, mice that developed nephritis displayed higher BUN levels compared with those without nephritis and controls (Supplemental Figure 1E). No significant differences were observed in the urine protein/creatinine ratio (UPCR) among experimental groups or between nephritis and nonnephritis mice (Supplemental Figure 1, C and F).

### Inflammatory repertoire

#### Inflammatory immune cell infiltration in kidney tissue.

**Figure 1:**
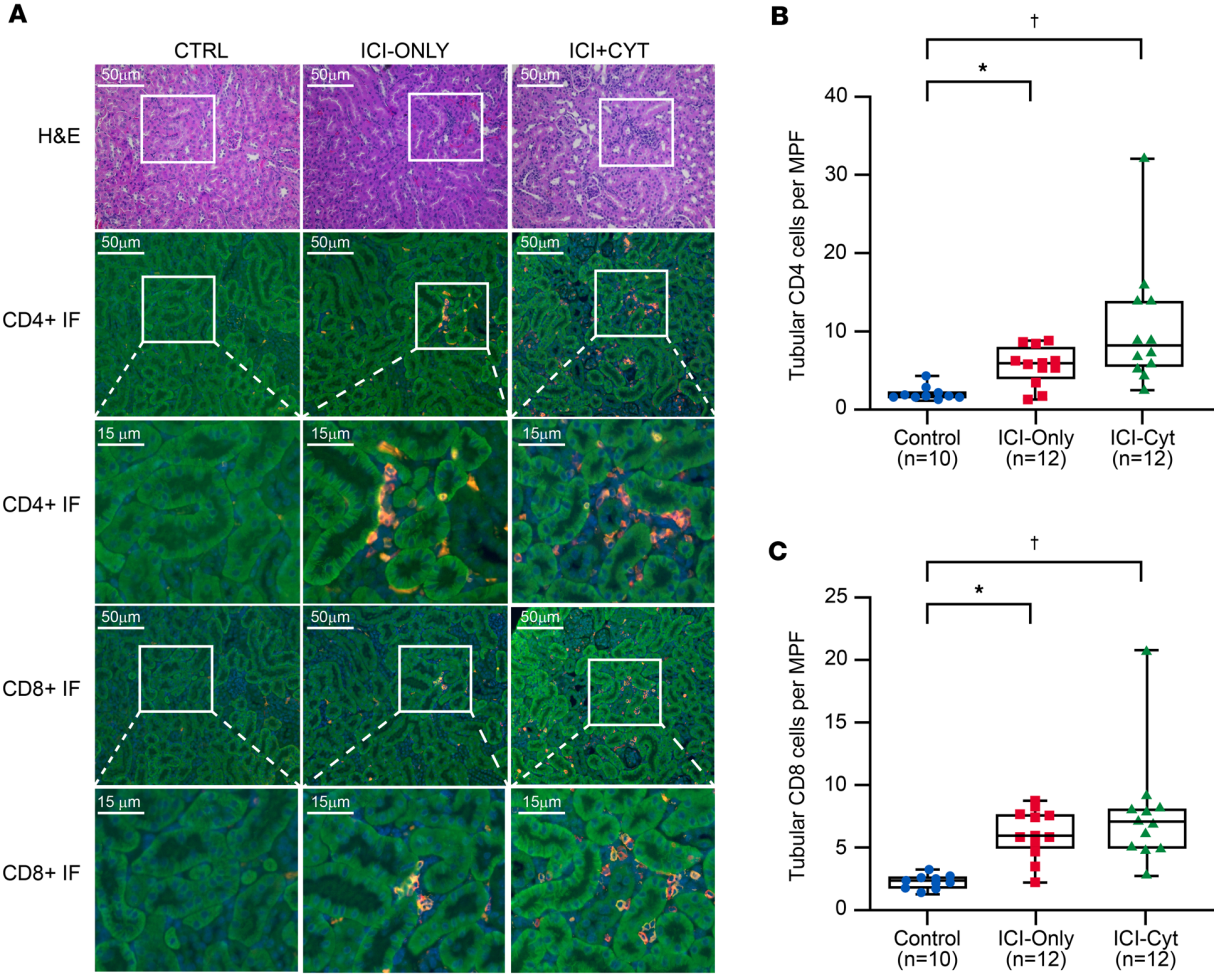
CD4^+^ and CD8^+^ tubular T cell number by immune checkpoint inhibitor treatment regimen. (**A**) Representative images of H&E staining (top row) and CD4^+^/CD8^+^ immunofluorescence (IF) staining (middle and bottom rows, respectively). White square highlight interstitial areas of interest in both H&E and IF images. (**B** and **C**) Quantification of tubular CD4^+^/CD8^+^ cells per medium power field (MPF) across groups. *P* values derived from Kruskal-Wallis followed by Dunn’s multiple-comparison test for post hoc analysis. Experiment was done once. *P < 0.05 for group ICI-Only (median: 5.96 CD4^+^ cells, 5.85 CD8^+^ cells) versus Control group (median: 1.9 CD4^+^ cells, 2.25 CD8^+^ cells) in post hoc test adjusted for multiple comparisons. ^†^*P* < 0.05 for group ICI-Cyt (median: 8.2 CD4^+^ cells, 7.05 CD8^+^ cells) versus Control group (median: 1.9 CD4^+^ cells, 2.25 CD8^+^ cells) in post hoc test adjusted for multiple comparisons. Scale bars: 50 μm and 15 μm as indicated.

**Figure 2:**
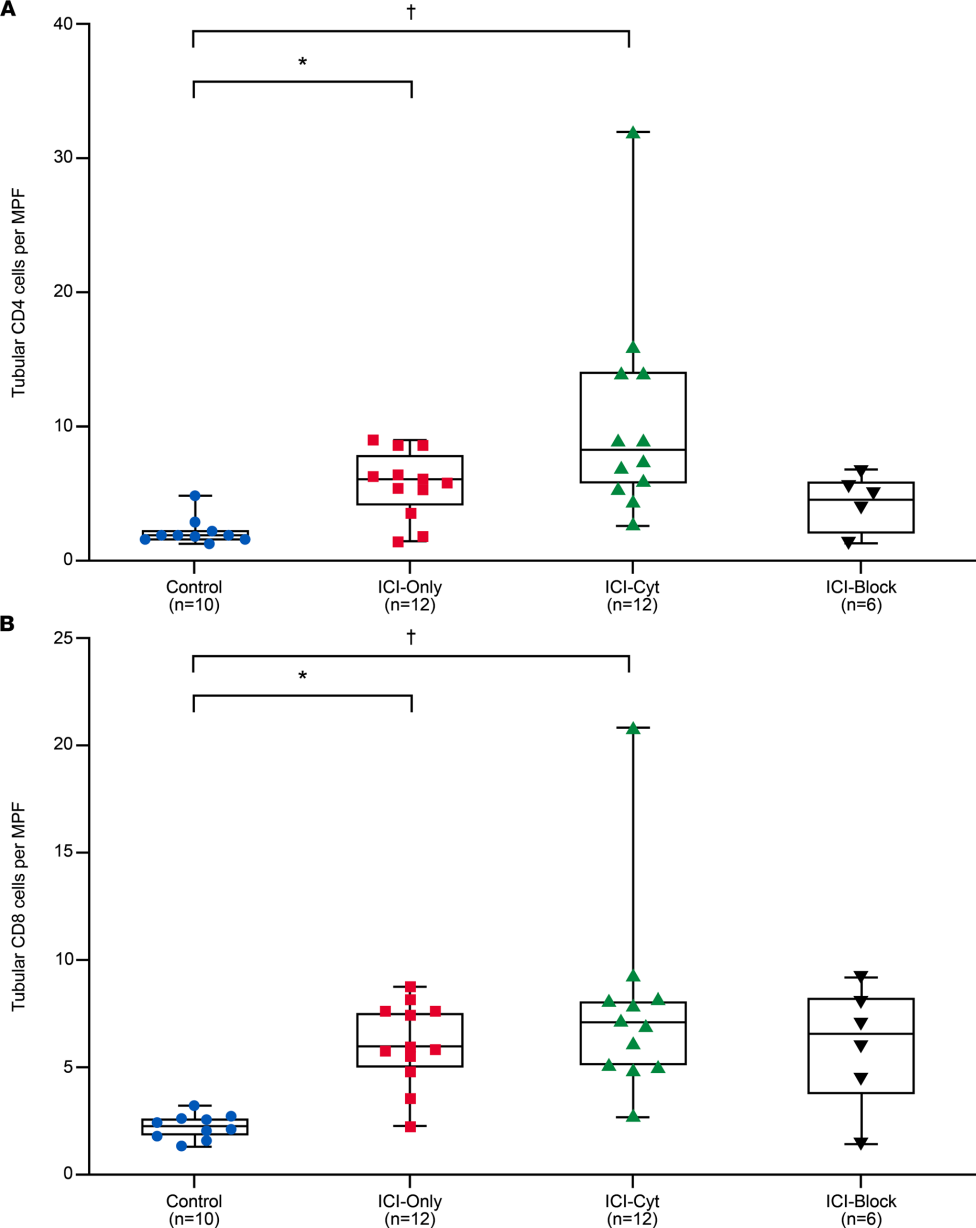
CD4^+^ and CD8^+^ tubular T cell number by immune checkpoint inhibitor treatment group and TNF-α blockade group. (**A** and **B**) Quantification of tubular CD4^+^ and CD8^+^ cells per medium power field (MPF) across treatment groups. *P* values derived from Kruskal-Wallis followed by Dunn’s multiple-comparison test for post hoc analysis. Experiment was done once. *P < 0.05 for group ICI-Only versus Control group in post hoc test adjusted for multiple comparisons. ^†^*P* < 0.05 for group ICI-Cyt versus Control group in post hoc test adjusted for multiple comparisons.

Glomerular immunofluorescence (IF) analysis revealed a higher number of CD4^+^ cells per glomerulus in the ICI-Cyt group (*P* = 0.03) (Supplemental Figure 2, A and B) while CD8^+^ cells showed an upward trend (*P* = 0.06) in the same group when compared with controls (Supplemental Figure 2, C and D). Tubular analysis demonstrated an increased presence of CD4^+^ cells per medium power field (MPF) in both ICI-Cyt group (*P* = 0.0001), and ICI-Only group (*P* = 0.03) relative to control group (Figure 1, A and B). Similarly, the number of CD8^+^ cells was significantly higher in the ICI-Cyt (*P* = 0.0001) and ICI-Only (*P* = 0.002) groups compared with controls (Figure 1, A and C). When treatment groups were compared, animals receiving concomitant TNF‑α blockade exhibited reduced CD4+ and CD8+ tubular T cell infiltration compared with ICI-treated groups, reaching levels comparable with controls (Figure 2, A and B).

The Nephritis group had significantly higher tubular CD4^+^ (*P* ≤ 0.001), and CD8^+^ (*P* ≤ 0.0001) cells compared with controls (Supplemental Figure 3, C and D). Although there was increased inflammation compared with the ICI-treated Non-Nephritis group, the difference was not statistically significant (Supplemental Figure 3, C and D).

#### Tubular TNF-α expression in kidney tissue.

**Figure 3:**
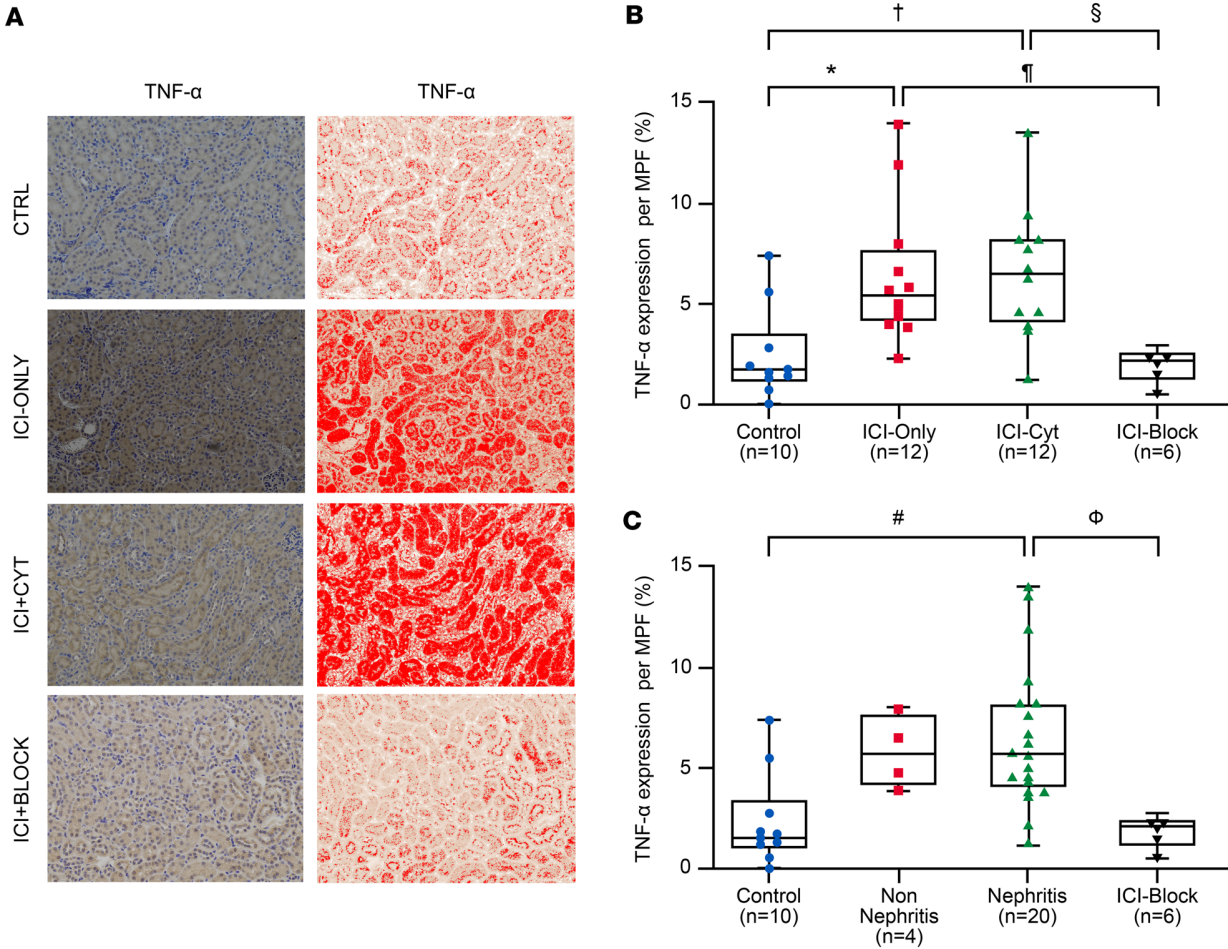
TNF-α expression in renal tubular compartments during immune checkpoint inhibition and TNF-α blockade. (**A**) Representative immunohistochemical (IHC) images showing TNF-α staining in kidney tubules across treatment groups. Left column displays TNF-α expression in brown, while right column presents red-enhanced images generated using ImageJ (NIH) highlight staining intensity. Original magnification, ×200. (**B**) TNF-α expression percentage per medium power field (MPF) in treatment groups. (**C**) TNF-α expression percentage per MPF in nephritis status groups. *P* values derived from Kruskal-Wallis followed by Dunn’s multiple-comparison test for post hoc analysis. Experiments were done once. Treatment groups: *P < 0.05 for group ICI-Only versus Control in post hoc test adjusted for multiple comparisons. ^¶^*P*<0.05 for group ICI-Only versus ICI-Block in post hoc test adjusted for multiple comparisons. ^†^*P* < 0.05 for group ICI-Cyt versus Control in post hoc test adjusted for multiple comparisons. §*P* < 0.05 for group ICI-Cyt versus ICI-Block in post hoc test adjusted for multiple comparisons. Nephritis groups: ^#^*P* < 0.05 for group Nephritis versus Control in post hoc test adjusted for multiple comparisons. ^Ф^*P* < 0.05 for group Nephritis versus ICI-Block in post hoc test adjusted for multiple comparisons.

Both groups treated with ICI therapy, ICI-Cyt and ICI-Only, showed enhanced TNF-α expression in the kidney tissue compared with controls (Figure 3, A and B). When treatment groups were compared, animals receiving concomitant TNF‑α blockade exhibited reduced CD4+ and CD8+ tubular T cell infiltration compared with ICI-treated groups, reaching levels comparable with controls (Figure 2, A and B). Similarly, the Nephritis group presented with higher expression of TNF-α when compared with controls (Figure 3C).

#### Cytokine profile in whole kidney tissue.

Qualitative cytokine array screening suggested increased renal expression of TNF-α, IFN-γ, and CXCL9 in ICI-treated mice compared with controls, particularly in the ICI-Cyt group (Supplemental Figure 4A). Based on these findings, absolute cytokine concentrations were quantified by ELISA.

TNF-α concentrations in whole kidney tissue were significantly higher in the ICI-Cyt group compared with control mice (Supplemental Figure 4B), with a trend toward higher levels compared with the ICI-Block group (*P* = 0.05) (Supplemental Figure 4B). By nephritis status, TNF-α levels were significantly elevated in mice with nephritis compared with all other groups (Supplemental Figure 4E). Across treatment groups, IFN-γ concentrations did not differ; however, IFN-γ levels tended to be higher in the ICI-Only group compared with controls (*P* = 0.05; Supplemental Figure 4C). In contrast, stratification by nephritis status revealed significantly higher IFN-γ levels in nephritic kidneys compared with both control and ICI-Block groups (Supplemental Figure 4F). CXCL-9 concentrations were higher in the ICI-Cyt group compared with control mice (Supplemental Figure 4D). When analyzed by nephritis status, CXCL-9 levels exhibited an upward trend in nephritic kidneys compared with controls (*P* = 0.05) (Supplemental Figure 4G).

### Plasma cytokine profile

Both treatment groups, ICI-Only and ICI-Cyt, showed elevated concentrations of IL-6, MCP-1, and TNF-α compared with the control group (Supplemental Table 2). Within the ICI-treated groups, the Nephritis group exhibited higher levels of IL-6, MCP-1, and TNF-α relative to controls. Similarly, the Non-Nephritis group showed increased MCP-1 and TNF-α levels as compared with the control group (Supplemental Table 2).

### Preventive effect of TNF-α blockade during ICI therapy combined with costimulation by inflammatory cytokines

Concomitant TNF-α blockade during ICI therapy, combined with costimulation by TNF-α and IFN-γ, resulted in attenuation of few renal parameters. The ICI-Block group (treated with ICIs, cytokines pump, and TNF-α blockage) presented with significantly lower BUN levels compared with both ICI-Only and ICI-Cyt groups (Supplemental Figure 5B), as well as the Nephritis group (Supplemental Figure 5E).

Although TNF-α blockade did not fully prevent CD4^+^ and CD8^+^ infiltration in the kidney, it reduced it to levels comparable with the control group not treated with ICI therapy (Figure 2, A and B). Similarly, TNF-α expression in the kidney tissue was reduced in the ICI-Block group compared with ICI-Only and ICI-Cyt (Figure 3, A and B) with comparable results observed relative to the Nephritis status (Figure 3C). Additionally, plasma concentrations of TNF-α and MCP-1 were lower in the ICI-Block group compared with the ICI-Cyt, ICI-Only, and Nephritis groups, confirming effective systemic blockade of the TNF-α pathway (Table 2).

### Single-cell RNA-seq reveals immune cell heterogeneity and differential T cell responses in ICI-AIN

**Figure 4:**
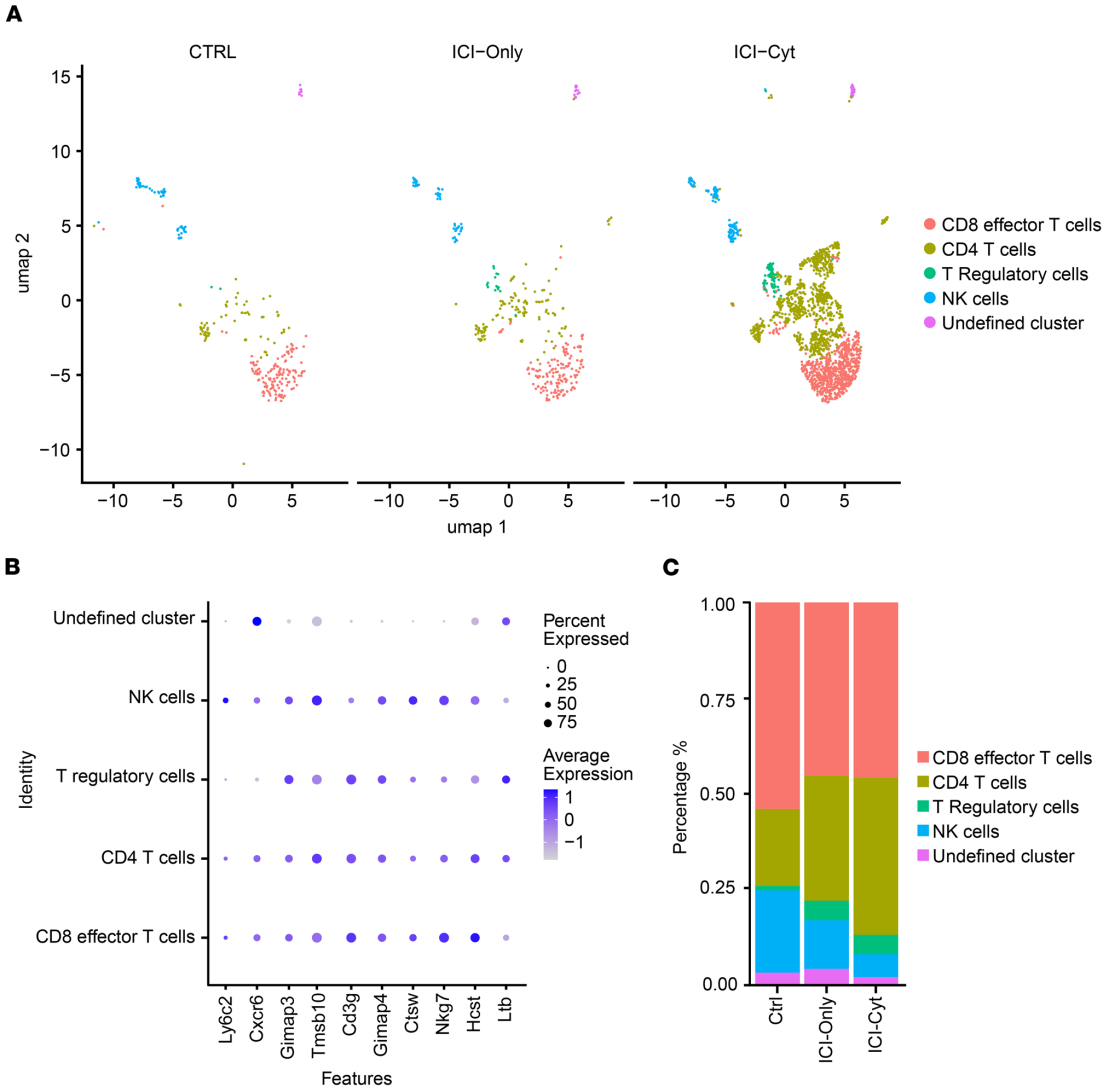
Single-cell transcriptomic profiling of kidney tissue across immune checkpoint inhibitor treatment groups. (**A**) T_NK cluster UMAP by treatment regimen. (**B**) Dot plot of genes used to subcluster. (**C**) Relative abundance percentage of cell types in cluster T_NK by treatment group.

**Figure 5:**
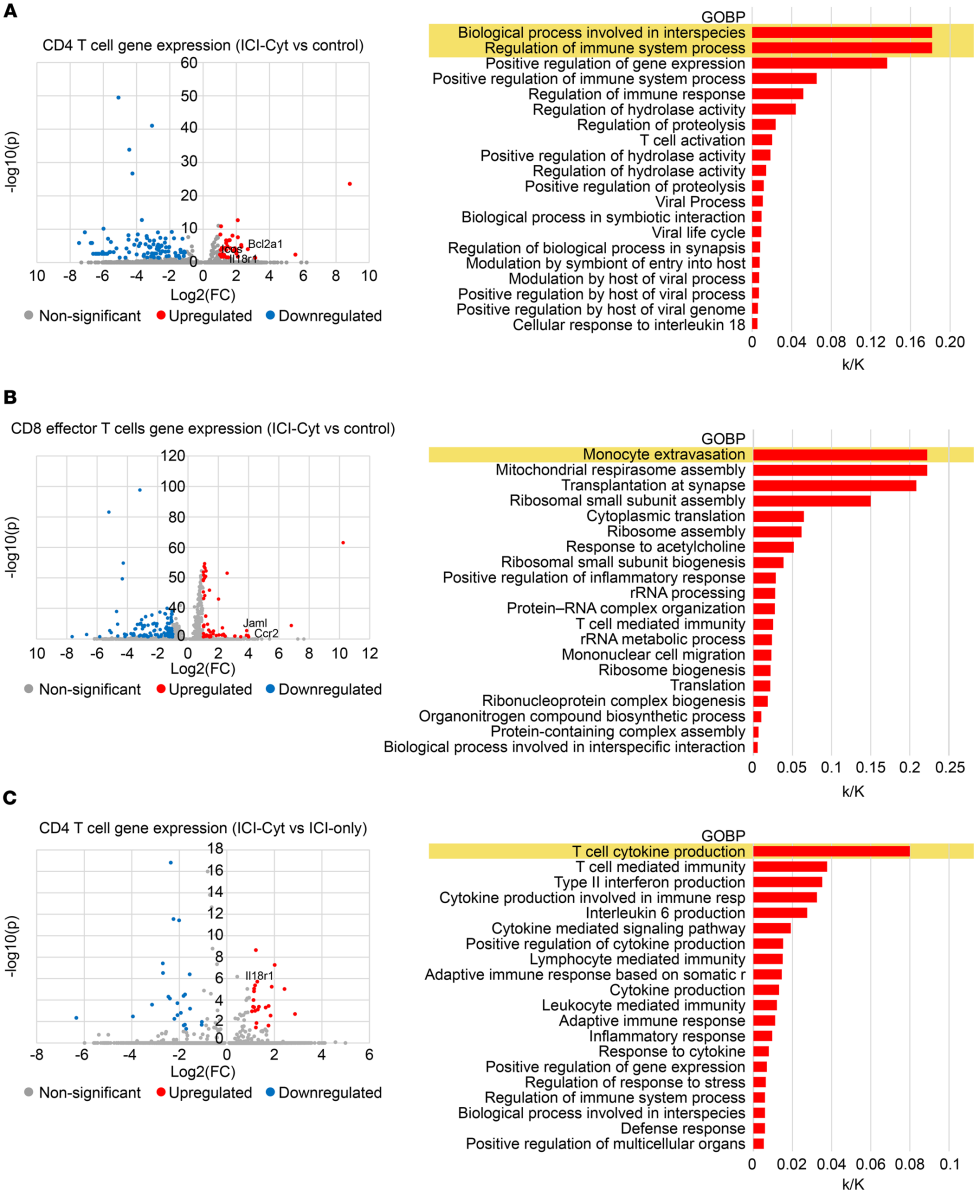
Biological analysis of gene expression for upregulated genes. (**A** and **B**) Volcano plot for differential gene expression and upregulated genes biological process analysis from gene set enrichment analysis (GSEA) for ICI-Cyt against Control CD4^+^ T cells and CD8^+^ effector cells, respectively. (**C**) Volcano plot for differential gene expression and upregulated genes biological process analysis from GSEA for ICI-Cyt against ICI-Only CD4^+^ T cells.

Cells passing quality control were analyzed (Supplemental Figure 6A). After filtering and normalization of data, dot plot analysis using canonical markers from literature identified 20 cell clusters, corresponding to 12 distinct cell types (Supplemental Figure 6, B and C). Due to their critical role in the development of ICI-AIN and as targets of ICIs, NKT cell cluster was selected for in-depth analysis. Following the same cluster definition methodology and using resolution of 0.1, five cell types and clusters were identified: CD8^+^ effector T cells, CD4^+^ T cells, Tregs, NK T cells, and an undefined cluster (Figure 4, A and B). Relative abundance analysis of annotated cell types revealed increased proportions of CD4^+^ T cells and Tregs in both ICI-Only and ICI-Cyt groups compared with controls, whereas CD8^+^ effector T cells and NK cells were decreased (Figure 4C). Gene set enrichment analysis (GSEA) comparing ICI-Cyt with Controls revealed upregulation in CD4^+^ T cell genes involved in interspecies interactions and immune regulation (Figure 5A), whereas CD8^+^ effector T cell genes were enriched for monocyte extravasation pathways (Figure 5B). Comparing ICI-Cyt with ICI-Only, CD4^+^ T cells showed increased expression of genes related to T cell cytokine production (Figure 5C). BCL2 Related Protein A1 (*Bcl2a1*), Inducible T Cell Costimulator (*Icos*), IL-18 Receptor 1 (*Il18r1*), C-C Motif Chemokine Receptor 2 (*Ccr2*), and Junction Adhesion Molecule Like (*Jaml*) were identified as key genes within highest k/K gene sets (Supplemental Table 3). A similar analysis was conducted on the downregulated genes; however, no immunological genes were found to be predominantly affected (Supplemental Figure 7).

## Discussion

The advent of ICIs has revolutionized cancer therapy over the past decade, demonstrating broad efficacy across a wide range of malignancies. However, this success has been tempered by the emergence of irAEs, particularly in organs such as the kidney (1). In this study, we utilized a humanized mouse model to investigate the underlying mechanisms and severity of kidney irAEs, aiming to provide deeper insight into their pathogenesis.

The study provides significant insights into the mechanisms underlying ICI-AIN and the potential therapeutic benefits of TNF-α blockade. Our findings demonstrate that ICI therapy, particularly when combined with proinflammatory cytokines such as TNF-α and IFN-γ during a period of 4–8 weeks, leads to early signs of renal dysfunction, immune cell infiltration, and elevated systemic and kidney tissue levels of cytokines. These results align with previous studies that have shown upregulation of inflammatory signatures in kidney biopsies from patients with ICI-AIN (10, 12).

To date, Hu PD-1/PD-L1 immunocompetent mice have not been used to evaluate the renal effects of ICIs in vivo. The introduction of a humanized chimeric mouse model in this study allowed for a more physiologically relevant replication of ICI-AIN compared with immune-deficient models, which lack the ability to mount normal immune responses, and allowed for a more accurate assessment of ICI-induced immune activation and tissue-specific toxicity (17). Additionally, humanized mouse models help overcome species-specific differences in immune checkpoint biology, such as the reduced inhibitory efficiency of murine PD-1, offering a more accurate platform to assess both therapeutic efficacy and toxicity of ICI therapies (18).

Despite a relatively short duration of ICI treatment (4–8 weeks), treated groups showed an already significant increase in a few biochemical renal parameters related to renal dysfunction, which in human serological parameters of AKI occurs between 14 and 16 weeks (7). The elevated plasma creatinine and BUN levels observed in ICI-treated groups, as well as similar findings when groups were stratified by nephritis status, reflect comparable findings also seen in patients experiencing renal irAEs during ICI therapy (8).

Histological analyses of human kidney biopsies have consistently shown that the predominant pattern of ICI-associated nephrotoxicity is interstitial or tubulointerstitial nephritis (8, 19). In our humanized model, ICI-treated groups exhibited increased CD4^+^ and CD8^+^ T cell infiltration within the tubular compartment. Similar to human ICI-associated nephritis, this infiltrate demonstrated a clear predominance of CD4^+^ T cells, closely reflecting the immunologic profile observed in human kidney tissue (10). Although the overall tissue inflammation grading assessed by the veterinary pathologist was predominantly mild to moderate, approximately 40% of mice in the ICI-Cyt group exhibited more severe inflammation after 8 weeks of treatment. Therefore, extending the treatment duration would likely exacerbate these findings. Interestingly, and consistent with the heterogeneity observed in human cases, not all ICI-treated mice developed significant nephritis: 4 of 24 ICI-treated animals showed no evidence of either glomerular or tubular inflammation. These mice were exclusively from the ICI-Only group, underscoring the role of cytokine-driven immune activation in promoting nephritis and reinforcing the translational relevance of this model.

Interstitial nephritis is widely recognized as a cytokine-mediated hypersensitivity reaction driven by specific T cell subsets (20). In human ICI-AIN, kidney biopsies typically reveal a lymphocytic infiltrate predominantly composed of CD4^+^ T cells (10, 21, 22). Consistent with these observations, IF microscopy confirmed a similar pattern of increased CD4^+^ T cell infiltration within the tubulointerstitial compartment. Notably, we also observed a significant increase in CD8^+^ T cells, which may reflect the well-established role of CD4^+^ cells in enhancing CD8^+^ mediated antitumor responses (23). While ICI therapy typically affects the tubulointerstitial compartment, glomerular involvement has also been reported. In our model, subtle glomerular infiltration was observed, potentially linked to anti-CTLA-4 exposure, which has been associated with glomerular damage (podocytopathy, membranous nephropathy, and thrombotic microangiopathy) (7, 24).

Our results also highlight the systemic effect of ICI therapy, as evidenced by elevated plasma levels of IL-6, MCP-1, and TNF-α in ICI-treated mice. These cytokines are known to play crucial roles in inflammatory responses, and their elevated levels further confirm the proinflammatory environment induced by ICI therapy. Interestingly, the TNF-α blockade group showed significantly lower levels of these cytokines, especially TNF-α (in both blood and kidney tissue) and MCP-1 (in blood). This reduction restored the kidney inflammatory profile to levels comparable with controls, suggesting that TNF-α inhibition can partially mitigate the systemic inflammatory response associated with ICI therapy. This highlights the relevance of this cytokine in the pathophysiology of immune-related kidney injury (25, 26). Our findings highlight the potential role of TNF-α blockade as a treatment option for refractory ICI-AIN and as a targeted strategy to enable safe ICI rechallenge in patients with prior ICI-AIN and no alternative anticancer options, rather than routine prophylaxis (13). By blocking the TNF-α pathway, renal irAEs may be prevented or their severity reduced, allowing immunotherapy to continue or be maintained with fewer interruptions or with a very low dose of corticosteroid therapy. Direct TNF-α blockade also reduced activation of other inflammatory pathways within kidney tissue — more significantly, the IFN-γ pathway, as shown in our renal tissue cytokine profile, which has been implicated as a key driver in the development of renal irAEs (12). In a cancer context, TNF-α exerts dual roles: supporting cytotoxic T cell–mediated tumor cell death and promoting tumor growth through angiogenesis. TNF-α blockade have shown positive outcomes to overcame resistance to anti–PD-1 therapy in mouse models. Human studies show more conflicting results, but TNF-α blockade is generally safe, with most patients maintaining stable disease or achieving tumor regression, though larger studies are needed to confirm oncologic outcomes (13, 15).

The single-cell RNA-seq analysis provided deeper insights into the cellular and molecular changes induced by ICI therapy. Significant transcriptional changes existed, including an increased relative abundance of CD4^+^ T cells in treatment groups, consistent with their role as immunological targets of checkpoint blockade. This finding is particularly relevant in the context of CTLA-4 inhibition, where the balance between activation and suppression is critically modulated. Tregs are crucial in this setting, as they help to maintain immune homeostasis and prevent autoimmunity. Tregs achieve this, in part, by limiting the ability of antigen-presenting cells (APCs) to deliver costimulatory signals via CD80 and CD86 to effector T cells by expressing high levels of the coinhibitory receptor CTLA-4, which outcompetes the effector T cell costimulatory receptor CD28 for binding to CD80 and CD86 and depleting them from the APC surface (27). This mechanism underscores the dual role of CD4^+^ T cells in ICI-induced nephrotoxicity; while effector CD4^+^ cells may drive inflammation, Tregs attempt to mitigate. Therefore, the presence and abundance of Tregs become especially important in counterbalancing the proinflammatory environment induced by CTLA-4 blockade—which, if unregulated, may exacerbate tissue damage by dismantling critical checkpoints of immune regulation.

In addition to these cellular dynamics, our study also identified key genes such as *Bcl2a1*, *Icos*, *Il18r1*, *Ccr2*, and *Jaml* within the NKT cell cluster highlighting them as potential targets for therapeutic intervention. The increased proportions of CD4^+^ T cells and Tregs in ICI-treated groups, along with the upregulation of genes involved in immune regulation and cytokine production, suggest that these cells play a pivotal role in the development of ICI-AIN.

This study provides important insights into the early inflammatory mechanisms associated with ICI-induced kidney injury. However, a few limitations should be acknowledged. First, the 8-week follow-up captures only the initial inflammatory phase and may have been too short to detect significant biochemical changes, such as elevated serum creatinine. Additionally, the lower body weight of ICI-treated mice may have contributed to lower creatinine levels minimizing differences in serum creatine levels compared with control mice. Extending the observation period in future studies could better characterize the progression and/or resolution of injury. Second, the use of relatively young mice may influence the susceptibility to AKI, as aging kidneys are known to exhibit reduced protective mechanisms and increased vulnerability to injury (25, 26). Including older animals in the future may clarify age-related differences in renal response. Lastly, a key limitation of the current model is the absence of a tumor component, which is central to the clinical context of ICI therapy. Incorporating a tumor to be treated would enhance the translational relevance of the model and allow for a more accurate evaluation of the balance between therapeutic efficacy and immune-related toxicity.

Despite these limitations, the model demonstrates several strengths that lay a solid foundation for future investigations and refinement. The use of immunocompetent mice enables direct evaluation of the immune response to ICIs, avoiding limitations of immunodeficient or xenograft models. A major innovation is the addition of a proinflammatory cytokine background and evaluation of cytokine blockade, which better mimics the complex immune environment seen in patients. Notably, all mice in the ICI-Cyt group developed some degree of interstitial inflammation, compared with 67% in the ICI-Only group. Moreover, transcriptomic immune profiling revealed stronger gene expression changes in cytokine-exposed mice, further supporting the model’s relevance for studying ICI-associated AIN.

In conclusion, our study using a humanized, fully immunocompetent mouse model underscores its value in replicating the complex immune dynamics of ICI-AIN. The findings suggest that TNF-α blockade may be a promising therapeutic strategy, not only for refractory cases but also as a preventive approach during ICI rechallenge, aiming to reduce the risk or severity of recurrent ICI-AIN. This strategy could improve patient outcomes and lessen clinical management burden. Further research is warranted to validate these findings in clinical settings and to explore additional therapeutic targets identified through this model.

## Methods

### Sex as a biological variable.

Both male and female mice were included in each group of our study. Sex was not considered as a biological variable.

### Study sample.

Forty male and female humanized PD-1/PD-L1 mice aged 8–12 weeks, from Haidong Dong’s Laboratory (Mayo Clinic; Rochester, Minnesota, USA) were studied over 8 weeks (Supplemental Table 4) (17). They were housed at Mayo Clinic Rochester’s Department of Comparative Medicine (DCM) under controlled conditions (constant temperature, 12-hour light/dark cycle, tap water, and regular diet).

Humanized PD-1/PD-L1 mice were randomly assigned to 1 treatment group: Control (saline, *n* = 10), ICI-Only (ICIs, *n* = 12), ICI-Cyt (ICIs + IFN-γ/TNF-α, *n* = 12), or ICI-Block (ICIs + cytokines + TNF-α blockade, *n* = 6). (Supplemental Table 4). ICIs used were human pembrolizumab (anti–PD-1, MERCK) and anti–mouse CTLA4 Invivomab (BioXCell) via i.p. injection starting on day 3 of the study protocol. A total of 5 doses of dual therapy was administered every 3 days. The cytokine infusion consisted of a pump implant that continuously delivered TNF-α and IFN-γ for 30 days. The ICI-Block group received anti–mouse TNF-α in addition to ICIs and cytokines. The initial batch of mice was euthanized on day 30, and samples were collected for further analysis (Supplemental Table 5). Subsequently, the remaining mice received an additional 5 doses of 4 mg/kg of pembrolizumab and were euthanized on day 60.

### Biochemical analysis.

Plasma creatinine (Arbor Assays, KB02-H1), Blood Urea Nitrogen (BUN) (BioAssay Systems, DIUR-100), urine creatinine (Arbor Assays, K002-H1), and urine protein levels (Bio-Rad, 50000201) were determined using the Synergy Mx Multi-Mode Microplate Reader (BioTek Instruments) following manufacturer’s instructions (Supplemental Methods).

### Histological analysis of renal morphology and inflammation.

Kidneys were sagittally dissected, fixed, embedded in paraffin, sectioned into 5 μm, and stained with H&E for histopathological examination and grading (see Supplemental Methods) by a veterinarian pathologist at the Pathology Research Core at Mayo Clinic, Scottsdale, Arizona (see Supplemental Methods).

IF and IHC staining were performed using the Leica Bond RX stainer. IF sections were stained with 2 antibody panels (CD4^+^ and CD8^+^, described in detail in Supplemental Methods and Supplemental Table 6) (28). IHC sections were stained with rabbit polyclonal TNF-α (Novus, NB600-587) according to manufacturer’s instructions (described in detail in Supplemental Methods).

One slide per animal was analyzed. Using a 20× objective lens, 20 random photographs were examined (10 images of cortical glomeruli and 10 of proximal and distal tubules). For IF, images were captured using the Axio Imager M2 microscope (ZEISS), while IHC images were obtained with the Nikon C-TEP3 microscope. Cell quantification was performed by direct manual counting under a fluorescence microscope, while the percentage area of TNF-α expression was determined using Fiji (Image J software), version 1.54g (29, 30). In both cases, an average value for glomeruli and for tubules was obtained for each slide.

### Cytokine quantification.

We used MILLIPLEX Mouse Cytokine/Chemokine Magnetic Bead Panel (Millipore, MCYTOMAG-70K, Darmstadt, Germany) to test plasma TNF-α, IL-6, and MCP-1. Readings were done with Luminex MAGPIX.

### Single-cell RNA-seq analysis.

Live single cells were isolated from kidney samples at the time of sacrifice. All samples were subjected to single-cell library preparation using Chromium Next GEM Single Cell 3′ Kit v3.1 (10X Genomics, Pleasanton, CA) (31). Libraries were sequenced using NovaSeq X Plus (Illumina, San Diego, CA) at 50,000 fragment reads per cell. Primary and secondary analysis were made following 10x Genomics protocol; more detailed description can be found in Supplemental Methods. Functional clustering analysis of differentially expressed genes was done using GSEA software (www.gsea-msigdb.org) by looking for overlay MSigDB database; only the top 20 biological process gene sets ranked by k/K ratio (# Genes in Overlap/ # Genes in Gene Set) were considered (32–35).

### Statistics.

Data were analyzed in JMP (JMP Statistical Discovery LLC), and respective graphs were obtained using GraphPad Prism version 10.4.2. For all figures, data with error bars are presented as box-and-whisker plots showing the median, interquartile range, and whiskers indicating the minimum and maximum values. Individual data points from each animal are overlaid on the plots. Normal distribution was determined by Shapiro-Wilk test. For normally distributed quantitative variables, we used 2-tailed Student’s *t* test and 1-way ANOVA followed by Tukey’s test for post hoc analysis. For nonnormally distributed variables, Wilcoxon rank sum test and Kruskal-Wallis test followed by Dunn’s test for post hoc analysis were used. *P* < 0.05 were considered statistically significant.

### Study approval.

All animal procedures were approved by the IACUC under protocol number A00007396-23. All experiments were conducted in accordance with the guidelines and standards established by the DCM at Mayo Clinic.

### Data availability.

All the data generated or analyzed in our study are available as supplemental files. Single-cell mRNA-seq samples generated in this study are available in Gene Expression Omnibus (GEO) database under accession number GSE296138. Values for all data points in graphs are reported in the Supporting Data Values file.

## Author contributions

SMH did the concept and design. All authors did acquisition or analysis, or interpretation of data. Drafting of the manuscript was done by VDCN, CNC, OAR, and SMH. All authors did critical revision of the manuscript for important intellectual content. Statistical analysis was performed by VDCN, OAR, and SMH. Preparation of figures and tables was done by VDCN, CNC, OAR, and SMH. VDCN, CNC, OAR, JEJJ, LEG, SZ, Xiaoyan Li, JBH, MFR, JH, Xiaogang Li, HD, AE, and SMH approved the definitive version of the manuscript, and all are accountable for all aspects of the submitted work.

## Conflict of interest

The authors have declared that no conflict of interest exists.

## Funding support

- Mayo CCaTS grant number UL1TR002377 and Mayo Clinic Fulk award (SMH).
- Mayo Clinic Foundation (MFR).

## Supplementary Material

Supplemental data

Supporting data values

## Figures and Tables

**Table 1:**
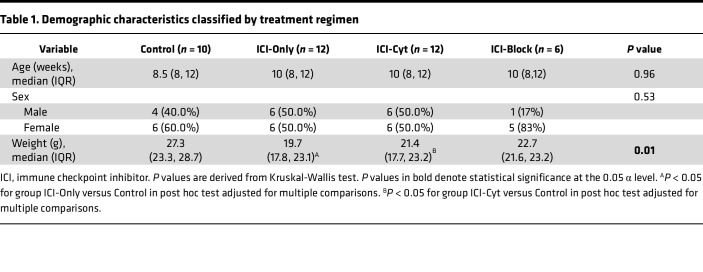
Demographic characteristics classified by treatment regimen

**Table 2:**
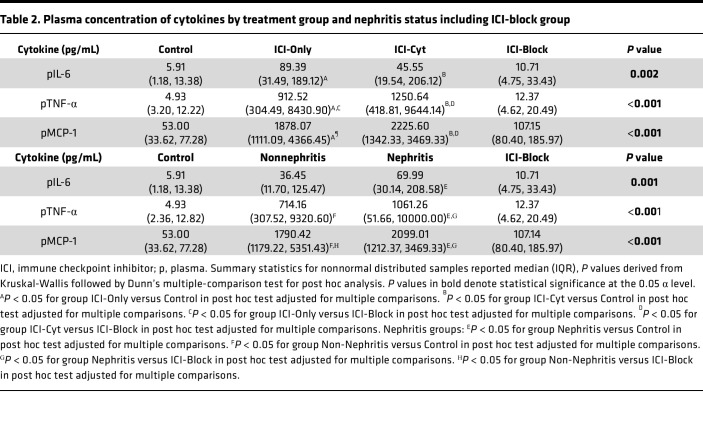
Plasma concentration of cytokines by treatment group and nephritis status including ICI-block group

## Version 1 03/05/2026

In-Press Preview

## Version 2 04/22/2026

Electronic publication

## References

[1] Postow MA (2018). Immune-related adverse events associated with immune checkpoint blockade. N Engl J Med.

[2] Brahmer JR (2012). Safety and activity of anti-PD-L1 antibody in patients with advanced cancer. N Engl J Med.

[3] Barbir EB (2024). Immune checkpoint inhibitor-associated nephritis-treatment standard. Nephrol Dial Transplant.

[4] Pardoll DM (2012). The blockade of immune checkpoints in cancer immunotherapy. Nat Rev Cancer.

[5] Herrmann SM, Perazella MA (2020). Immune checkpoint inhibitors and immune-related adverse renal events. Kidney Int Rep.

[6] Gupta S (2021). Acute kidney injury in patients treated with immune checkpoint inhibitors. J Immunother Cancer.

[7] Herrmann SM (2025). Diagnosis and management of immune checkpoint inhibitor-associated nephrotoxicity: a position statement from the American Society of Onco-nephrology. Kidney Int.

[8] Cortazar FB (2020). Clinical features and outcomes of immune checkpoint inhibitor-associated AKI: a multicenter study. J Am Soc Nephrol.

[9] García-Carro C (2022). Acute kidney injury as a risk factor for mortality in oncological patients receiving checkpoint inhibitors. Nephrol Dial Transplant.

[10] Farooqui N (2023). Cytokines and immune cell phenotype in acute kidney injury associated with immune checkpoint inhibitors. Kidney Int Rep.

[11] Long JP (2025). Urine proteomics defines an immune checkpoint-associated nephritis signature. J Immunother Cancer.

[12] Singh S Tertiary lymphoid structure signatures are associated with immune checkpoint inhibitor related acute interstitial nephritis. JCI Insight.

[13] Lin JS (2021). Infliximab for the treatment of patients with checkpoint inhibitor-associated acute tubular interstitial nephritis. Oncoimmunology.

[14] Barbir EB (2025). Biomarker-guided infliximab therapy for immune checkpoint inhibitor-induced acute interstitial nephritis. Nephrol Dial Transplant.

[15] Youssef N (2025). Infliximab for grade III or IV immune checkpoint inhibitor nephritis clinical and translational evidence. Kidney Int Rep.

[16] Zhou P (2024). Immune checkpoint inhibitors and acute kidney injury. Front Immunol.

[17] Barham W (2023). A novel humanized PD-1/PD-L1 mouse model permits direct comparison of antitumor immunity generated by food and drug administration-approved PD-1 and PD-L1 inhibitors. Immunohorizons.

[18] Tang Z, Veillette A (2025). Inhibitory immune checkpoints in cancer immunotherapy. Science Immunol.

[19] Xu LY (2023). Clinicopathological features of kidney injury related to immune checkpoint inhibitors: a systematic review. J Clin Med.

[20] Moledina DG (2019). Urine TNF-α and IL-9 for clinical diagnosis of acute interstitial nephritis. JCI Insight.

[21] Cortazar FB (2016). Clinicopathological features of acute kidney injury associated with immune checkpoint inhibitors. Kidney Int.

[22] Belliere J (2016). Acute interstitial nephritis related to immune checkpoint inhibitors. Br J Cancer.

[23] Topchyan P (2023). The role of CD4 T cell help in CD8 T cell differentiation and function during chronic infection and cancer. Immune Netw.

[24] Catalano M (2022). Immune checkpoint inhibitor induced nephrotoxicity: an ongoing challenge. Front Med (Lausanne).

[25] Bertrand F (2017). TNFα blockade overcomes resistance to anti–PD-1 in experimental melanoma. Nat Commun.

[26] Chen AY (2021). TNF in the era of immune checkpoint inhibitors: friend or foe?. Nat Rev Rheumatol.

[27] Wardell CM (2025). Harnessing the biology of regulatory T cells to treat disease. Nat Rev Drug Discov.

[28] Im K (2019). An introduction to performing immunofluorescence staining. Methods Mol Biol.

[29] Diem K (2015). Image analysis for accurately counting CD4+ and CD8+ T cells in human tissue. J Virol Methods.

[30] Stossi F, Singh PK (2023). Basic image analysis and manipulation in ImageJ/Fiji. Curr Protoc.

[31] https://cdn.10xgenomics.com/image/upload/v1722285481/support-documents/CG000315_ChromiumNextGEMSingleCell3__GeneExpression_v3.1_DualIndex__RevF.pdf.

[32] Liberzon A (2011). Molecular signatures database (MSigDB) 3.0. Bioinformatics.

[33] Liberzon A (2015). The molecular signatures database (MSigDB) hallmark gene set collection. Cell Syst.

[34] Castanza AS (2023). Extending support for mouse data in the molecular signatures database (MSigDB). Nat Methods.

[35] Subramanian A (2005). Gene set enrichment analysis: a knowledge-based approach for interpreting genome-wide expression profiles. Proc Natl Acad Sci U S A.

