# Supplementary material for: TNF-α blockade mitigates immune checkpoint–related nephritis in a humanized mouse model
Source: JCI Insight. 2026 Mar 5;11(8):e199694. doi: 10.1172/jci.insight.199694 (PMC13135400; doi:10.1172/jci.insight.199694)
Supplement: Supplemental data [file jciinsight-11-199694-s263.pdf]

SUPPLEMENTARY MATERIAL

Supplementary File (PDF)

Supplementary Methods

Supplementary Table S1. Demographic Characteristics by Nephritis Status

Supplementary Table S2. Plasma Concentration of Cytokines by Treatment Regimen and Nephritis Status

Supplementary Table S3. Biological Process Analysis

Supplementary Table S4. Treatment Regimen

Supplementary Table S5. Specimen Sources and Analysis

Supplementary Table S6. Immunofluorescence Antibody Panels

Supplementary Fig S1. Kidney Function Parameters Across Treatment Groups and Nephritis Status

Supplementary Fig S2. CD4+ and CD8+ Glomerular T Cell Number by Treatment

Supplementary Fig S3. CD4+ and CD8+ Glomerular and Tubular T Cell Number by Nephritis Status

Supplementary Fig. S4. Cytokine Concentration Profile in Kidney Tissue

Supplementary Fig. S5. Kidney Function Parameters Across Treatment Groups and Nephritis Status Including TNF-α Blockade Group

Supplementary Fig. S6. Treatment-Dependent Changes in Kidney Cell Clusters Revealed by Single-Cell Transcriptomics

Supplementary Fig. S7. Biological Analysis of Gene Expression for Downregulated Genes

References

Supplementary Methods

Biochemical Analysis

Serum creatinine was quantified using DetectX Serum Creatinine Kit from Arbor Assays (Cat. # KB02-H1). Prior to running in the assay all samples were centrifugated for 15 minutes at 14,000 rpm. Samples and standards (25 µl each) were pipetted into the respective wells of a 96-well plate (Cat. # X018-2EA). Then, 25 µl of Assay Diluent (Cat. # X017-6ML) was added to each well, followed by 100 µl of DetectX Creatinine Reagent (Cat. # C004-20ML). Optical density readings at 490 nm were conducted at 1 min and 30 min. Temporal changes in optical density were assessed by subtracting 1-minute values from 30-minutes values. These differences were plotted against creatinine concentrations of standards samples. A linear regression was generated using an equation to calculate the concentrations. The resulting values were then corrected based on each mouse's individual weight and the final data were analyzed using Prism.

Serum BUN was quantified using QuantiChrom Urea Assay Kit from BioAssay Systems (Cat. # DIUR-100). A total of 5 µl of each sample, standard and blank were transferred into wells of a clear bottom 96-well plate (Cat. # X003-2EA). Then, 200 µl of Working reagent combination was added to each well. Optical density readings at 520 nm were taken after 30 min. A linear regression was generated using the equation provided to calculate the urea concentrations and a conversion was applied to determine the BUN results.

For the urine creatinine measurement, all samples were diluted 1:20 with deionized water. Urine samples with creatinine concentrations exceeding the standard curve range were further diluted to ensure readings fell within the curve. A total of 50 µl of each sample, standard and blank were added into to respective wells of a 96-well plate (Cat. # X003-2EA), followed by the addition of 100 µl of DetectX Creatinine Reagent (Cat. # C004-20ML). The sealed plate underwent a 30-minute incubation before optical density readings were taken at 490 nm. A standard curve was generated, and the sample concentrations were calculated by multiplying the measured values by the dilution factor.

Urine protein was assessed using the Bio-Rad BCA protein assay with the albumin standard (Cat. #23210). A total of 10 µl of bladder urine sample and prepared standards were transferred into the respective wells of a 96-well plate followed by the addition of 190 µl 1x dye reagent to wells (1:4 dilution of Bio-Rad Category #500-0201). After a 10-minute incubation, an absorbance measure at 595 nm was performed. A linear regression curve was generated using the standards values to calculate concentrations.

Kidney Tissue Immunofluorescence Staining for CD4 and CD8 T Cells: Panel #1-CD4+CD3+ NBCe1A and Panel #2- CD8+CD3+ NBCe1A

Immunofluorescence (IF) staining was performed at the Pathology Research Core (Mayo Clinic, Rochester, MN) using the Leica Bond RX. Slides were stained using two different antibody panels. Panel #1 included rabbit CD4 (Abcam, cat# ab183685), rat CD3 (Abcam, cat# AB11089), and chicken NBCe1A (Aves Labs, generated by Dr. Romero lab) (1). Panel #2 included the same antibodies, but exchanged the CD4 for rabbit CD8 (Abcam, cat# ab209775). All slides were retrieved for 20 minutes using Epitope Retrieval 2 (EDTA; Leica) and blocked with 10% Goat Serum Block (Invitrogen) for 30 minutes; irrespective of the panel.

Both primary and secondary antibodies were diluted in Background Reducing Diluent (Dako) and incubated for 60 minutes. Rabbit CD4 was diluted at 1:1000, rabbit CD8 was diluted at 1:200; both rat CD3 and NBCe1A were diluted at 1:250. The following donkey secondary antibodies were used at a 1:250 dilution; anti-rabbit Cy3 (Jackson Immunoresearch, cat# 711-165-152), anti-chicken Alexa Fluor 488 (Jackson Immunoresearch, cat# 703-545-155), and anti-rat Alexa Fluor 647 (Jackson Immunoresearch, cat# 712-605-150). All secondary antibodies were first reconstituted in 400ul of distilled water and then diluted to its final dilution.

Slides were counterstained for 10 minutes at 1ug/ml using Hoechst 33342 (Invitrogen, cat# H3570). Once the immunofluorescence process was completed, slides were removed from the stainer and rinsed in tap water for one minute. ProLong Gold (Invitrogen, cat# P36934) mounting medium was used to permanently coverslip the slides.

Histological Evaluation of Inflammation and Nephritis Status Determination

The histopathology grading scale followed by our veterinarian pathologist is a semi-quantitative system used to assess tissue response based on cellular and structural changes observed under high-powered microscopy (approximately 400x). Nine histological parameters were evaluated: polymorphonuclear cells (PMNs), lymphocytes, plasma cells, macrophages, giant cells, necrosis, neovascularization, fibrosis, and fatty infiltrate. Each parameter was scored on a scale from 0 to 4. Due to the clinical relevance of inflammatory infiltrates and necrosis, the scores for PMNs, lymphocytes, plasma cells, macrophages, giant cells, and necrosis were multiplied by 2 to assign greater weight to these features. An average score per treatment group was calculated, and the degree of inflammation was determined by subtracting the control group average from the treatment group average. The inflammation grade was categorized as none (0.0–2.9), mild (3.0–8.9), moderate (9.0–15.0), or severe (>15) (2). Those with no inflammation after ICI treatment were classified as the Non-Nephritis group.

TNF-α Immunohistochemistry

Immunohistochemistry (IHC) staining was performed at the Pathology Research Core (Mayo Clinic, Rochester, MN) using the Leica Bond RX stainer (Leica). Tissue sections were cut at five microns, mounted on charged slides, and dried overnight. Slides stained with rabbit, polyclonal TNF-α (Novus, cat# NB600-587) were retrieved for 20 minutes using Epitope Retrieval 1 (Citrate; Leica) and incubated in Protein Block (Dako) for five minutes. The primary antibody was diluted at 1:600 in Background Reducing Diluent (Dako) and incubated for 15 minutes.

The detection system used was Polymer Refine Detection System (Leica). This system includes hydrogen peroxidase block, polymer reagent, and DAB. Immunostaining visualization was achieved by incubating slides for 10 minutes in DAB and DAB buffer (1:19 mixture) from the Bond Polymer Refine Detection System. Slides were counterstained for five minutes using a 1:1 mixture of Schmidt hematoxylin (Mayo DLMP Preparation and Processing Laboratory) and molecular biology grade water. Once the immunochemistry process was completed,

slides were removed from the stainer and rinsed in tap water for three minutes. Slides were dehydrated in increasing concentrations of ethyl alcohol and cleared in three changes of xylene prior to permanent coverslipping in xylene-based medium.

IFN-γ concentration from whole kidney tissue

Kidney tissue was homogenized and lysed using Cell Lysis Buffer (10X; Cell Signaling Technology, Cat. #9803) according to the manufacturer's instructions. For cytokine analyses, five samples per treatment group were analyzed. Nephritis status was determined prior to selection across all groups; therefore, in the ICI-only group all non-nephritis samples (n=4 of 12) were included, and five samples were randomly selected from the remaining nephritis samples (n=8). In the ICI-cyt group, all samples were nephritis, so no stratified selection by nephritis status was applicable. Lysates were clarified by centrifugation, and total protein content was determined for downstream analyses.

An exploratory cytokine screen was performed using a membrane-based antibody array (Proteome Profiler™ Mouse Cytokine Array Kit, Panel A, R&D Systems, Cat. #ARY006) according to the manufacturer's instructions. Kidney tissue lysates were incubated with a cocktail of biotinylated detection antibodies and applied to nitrocellulose membranes spotted in duplicate with capture antibodies against 40 mouse cytokines and chemokines. Bound proteins were detected using streptavidin–horseradish peroxidase and chemiluminescent reagents. Array images were used for qualitative and comparative visualization only and were not used for quantitative analysis.

Based on array screening results, absolute concentrations of IFN-γ, TNF-α and CXCL-9 in kidney tissue lysates were quantified using enzyme-linked immunosorbent assay (ELISA) kits from R&D Systems, following the manufacturers' protocols. Cytokine concentrations were calculated from standard curves, normalized to total protein content, and expressed as picograms per milligram of total protein (pg/mg).

Single Cell Processing

Whole live cells were isolated from kidney samples. Cell suspension was washed twice, resuspended in 1x PBS + 0.04% BSA, and immediately submitted to the Genome Analysis Core for Single Cell partitioning. The cells were counted and measured for viability using the Vi-Cell XR Cell Viability Analyzer (Beckman-Coulter). The barcoded Gel Beads were thawed from -80C and the cDNA master mix was prepared according to the manufacture's instruction for Chromium Next GEM Single Cell 3' Kit v3.1 (10x Genomics) (3). Based on the desired number of cells to be captured for each sample, a volume of live cells was mixed with the cDNA master mix. A per sample concentration of 500,000 cells per milliliter or better is required for the standard targeted cell recovery of up to 10,000 cells. The stock concentration requirements would not change for higher cell recovery numbers. The cell suspension and master mix, thawed Gel Beads and partitioning oil were added to a Chromium Next GEM G chip. The filled chip was loaded into the Chromium Controller, where each sample was processed and the individual cells within the sample were partitioned into uniquely labeled GEMs (Gel Beads-In-Emulsion). The GEMs were collected from the chip and taken to the bench for reverse transcription, GEM dissolution, and cDNA clean-up. The resulting cDNA contains a pool of uniquely barcoded molecules. A portion of the cleaned and measured pooled cDNA continues to library construction, where standard Illumina sequencing primers and a unique sample index (Dual Index Kit TT-Set A; 10x Genomics) were added to each cDNA pool, creating gene expression libraries. All cDNA pools and resulting libraries were measured using Qubit High Sensitivity assays (Thermo Fisher Scientific) and Agilent Bioanalyzer High Sensitivity chips (Agilent).

Libraries were sequenced at 50,000 fragment reads per cell following Illumina's standard protocol using the Illumina NovaSeq X Plus™. The flow cells were sequenced as 100x2 paired end reads using NovaSeq X Plus sequencing reagents and software. All primary data was analyzed using 10x Genomics Cell Ranger and Loupe Browser software packages.

Secondary analysis was performed at Mayo Clinical Sequencing Core with 10X Genomics Platform (https://www.10xgenomics.com/). The fastq files were processed with Cell

Ranger v7 (10X Genomics) for quality control, read mapping and cell profiling; mouse genome reference mm10 was used. The output then analyzed with Seurat V5 (https://satijalab.org/seurat/articles/get_started_v5_new). Briefly, cells were qualified with 1) percentage of mitochondria less than 13%, 2) within the range of 500-2500 for read count, 3) within the range of 1300-6500 for feature count. Then SCTransform was used for normalization and HarmonyIntegration was used for sample batch correction. Resolution 0.4 was used for UMAP clustering. For cell type identification, top expressed genes from each cluster were taken and PBMC and Kidney marker genes were searched in HuBMAP database, ScType database (https://sctype.app/) and Park. J et al (4–7). For sub cell type identification of T and NK cells, first subset the T and NK cell populations from the full dataset. Then unsupervised clustering was performed on the subset cells using a resolution parameter of 0.1 to generate refined clusters. Subtype identities were assigned to each resulting cluster using canonical marker genes as defined by Balzer et al (8).

Supplementary Tables and Figures

Supplementary Table S1. Demographic Characteristics by Nephritis Status

Variable	Control (n=10)	Non-Nephritis (n=4)	Nephritis (n=20)	ICI-Block (n=6)	*P* value
**Age (weeks), median (IQR)**	8.5 (8, 12)	10 (8, 12)	10 (8,12)	10 (8, 12)	0.96
**Gender**					0.34
Male	4 (40.0%)	1 (25.0%)	11 (55.0%)	1 (17.0%)	
Female	6 (60.0%)	3 (75.0%)	9 (45.0%)	5 (83.0%)	
**Weight (g), median (IQR)**	27.3 (23.3, 28.7)	19.2 (17.7, 23.9)	20.3 (17.8, 23.2)#	22.7 (21.6, 23.2)	**0.01**

P-values are derived from Kruskal-Wallis test. *P*-values in bold denote statistical significance at the 0.05 alpha level.
# $p<0.05$ for group Nephritis vs Control in post-hoc test adjusted for multiple comparisons.

Supplementary Table S2. Plasma Concentration of Cytokines by Treatment Regimen and Nephritis Status

Cytokine (pg/mL)	Control	ICI-Only	ICI-Cyt	*P* value
**pIL-6**	5.91 (1.18, 13.38)	89.39 (31.49, 189.12)*	45.55 (19.54, 206.12)†	**0.003**
**pTNF-α**	4.93 (3.20, 12.22)	912.52 (304.49, 8430.90)*	1250.64 (418.81, 9644.14)†	**<0.001**
**pMCP-1**	53.00 (33.62, 77.28)	1878.07 (1111.09, 4366.45)*	2225.60 (1342.33, 3469.33)†	**0.0004**
**Cytokine** (pg/mL)	**Control**	**Non-nephritis**	**Nephritis**	***P* value**
**pIL-6**	5.91 (1.18, 13.38)	36.45 (11.70, 125.47)	69.99 (30.14, 208.58)#	**0.002**
**pTNF-α**	4.93 (2.36, 12.82)	714.16 (307.52, 9320.60)‡	1061.26 (51.66, 10000.00)#	**<0.0001**
**pMCP-1**	53.00 (33.62, 77.28)	1790.42 (1179.22, 5351.43)‡	2099.01 (1212.37, 3469.33)#	**0.0004**

Abbreviations: ICI, immune checkpoint inhibitor; p, plasma.
Summary statistics for non-normal distributed samples reported median (IQR), p-values derived from Kruskal-Wallis followed by Dunn's multiple comparison test for post-hoc analysis.
* $p<0.05$ for group ICI-Only vs Control in post-hoc test adjusted for multiple comparisons.
† $p<0.05$ for group ICI-Cyt vs Control in post-hoc test adjusted for multiple comparisons.
‡ $p< 0.05$ for group Non-Nephritis vs Control in post-hoc test adjusted for multiple comparisons.
# $p< 0.05$ for group Nephritis vs Control in post-hoc test adjusted for multiple comparisons.

Supplementary Table S3. Biological Process Analysis

Cell Type	Comparison	Biological Process	Description	Genes of interest
CD4+ T cell	ICI-Cyt vs Control	Interspecies interaction between organisms	Any process evolved to enable an interaction with an organism of a different species.	*Bcl2a1*<br>*Icos*<br>*Il18r1*
		Regulation of immune system process	Any process that modulates the frequency, rate, or extent of an immune system process.	
CD8+ effector T cell	ICI-Cyt vs Control	Monocyte Extravasation	The migration of a monocyte from the blood vessels into the surrounding tissue.	*Ccr2*<br>*Jaml*
CD4+ T cell	ICI-Cyt vs ICI-Only	T cell cytokine production	Any process that contributes to cytokine production by a T cell.	*Il18r1*

Abbreviations: BCL2 related protein A1 (Bcl2a1), Inducible T Cell Costimulator (Icos), Interleukin 18 Receptor 1 (Il18r1), C-C Motif Chemokine Receptor 2 (Ccr2), Junction Adhesion Molecule Like (Jaml).

Supplementary Table S4. Treatment Regimen

Mouse Type (N; Male (M), Female (F))	Cytokine Pump	Treatment Regimen	Treatment Duration (Weeks)
**Hu-PD-1/PD-L1 (26: 9M,17F)**	No pump	Vehicle (Saline)	**4**
		Pembrolizumab (20 mg/kg) + anti-CTLA-4 Invivomab (25 mg/kg) q3d x5 doses	
	Cytokine Pump (200 ng/day TNF-α and 100 units/gram IFN-γ)	Pembrolizumab (20 mg/kg) + anti-CTLA-4 Invivomab (25 mg/kg) q3d x5 doses + Cytokine Pump	
		Pembrolizumab (20 mg/kg) + anti-CTLA-4 Invivomab (25 mg/kg) q3d x5 doses + Cytokine Pump + anti-TNF-α 150 mg/kg	
**Hu-PD-1/PD-L1 (14: 8M,6F)**	No pump	Vehicle (Saline)	**8**
		First 4 weeks: Pembrolizumab (20 mg/kg) + anti-CTLA-4 Invivomab (25 mg/kg) q3d x5 doses<br>Second 4 weeks: Pembrolizumab (20 mg/kg)	

	Cytokine Pump (200 ng/day TNF-α and 100 units/gram IFN-γ)	First 4 weeks: Pembrolizumab (20 mg/kg) + anti-CTLA-4 Invivomab (25 mg/kg) q3d x5 doses + Cytokine Pump<br>Second 4 weeks: Pembrolizumab (20 mg/kg)	

TNF-α Cat. #410-MI-100/CF and INF- γ **Cat**. #485-MI-100/CF from R&D Systems. **Pembrolizumab (Keytudra)** from MERCK and **Invivomab** anti-mouse CTLA-4 Cat. #BE0032 from BioXCell Technology. **InVivoMAb** anti-mouse TNFα Cat. #BE0058 from BioXCell Technology.

Supplementary Table S5. Specimen Sources and Analysis

Sample Origin	Processing
**Urine (bladder)**	Protein, Creatinine.
**Blood (heart)**	Creatinine, BUN, Cytokine profile.
**Kidney**	Immunofluorescent microscopy (CD3, CD4, and CD8), and Brightfield microscopy (TNF-alpha and H&E).<br>Single cell analysis.

Abbreviations: BUN, Blood urea nitrogen; CD, Cluster of differentiation; TNF, Tumor necrosis factor; H&E, Hematoxylin and Eosin.

Supplementary Table S6. Immunofluorescence Antibody Panels

Panel #1	Panel #2
Rabbit CD4 (Abcam, cat# ab183685)	Rabbit CD8 (Abcam, cat# ab209775)
Rat CD3 (Abcam, cat# AB11089)	Rat CD3 (Abcam, cat# AB11089)
Chicken NBCe1A (Aves Labs, generated by Dr. Romero lab)	Chicken NBCe1A (Aves Labs, generated by Dr. Romero lab)
Primary and secondary antibodies were diluted in Background Reducing Diluent (Dako) and incubated for 1 hour.<br>Rabbit CD4 was diluted 1:1000, rabbit CD8 1:200, and both rat CD3 and NBCe1A 1:250.<br>Secondary antibodies (donkey anti-rabbit Cy3 (Jackson Immunoresearch, cat# 711-165-152), anti-chicken Alexa Fluor 488 (Jackson Immunoresearch, cat# 703-545-155), and anti-rat Alexa Fluor 647 647 (Jackson Immunoresearch, cat# 712-605-150)) were used at 1:250 dilution.	

Supplementary Fig S1. Kidney Function Parameters Across Treatment Groups and Nephritis Status

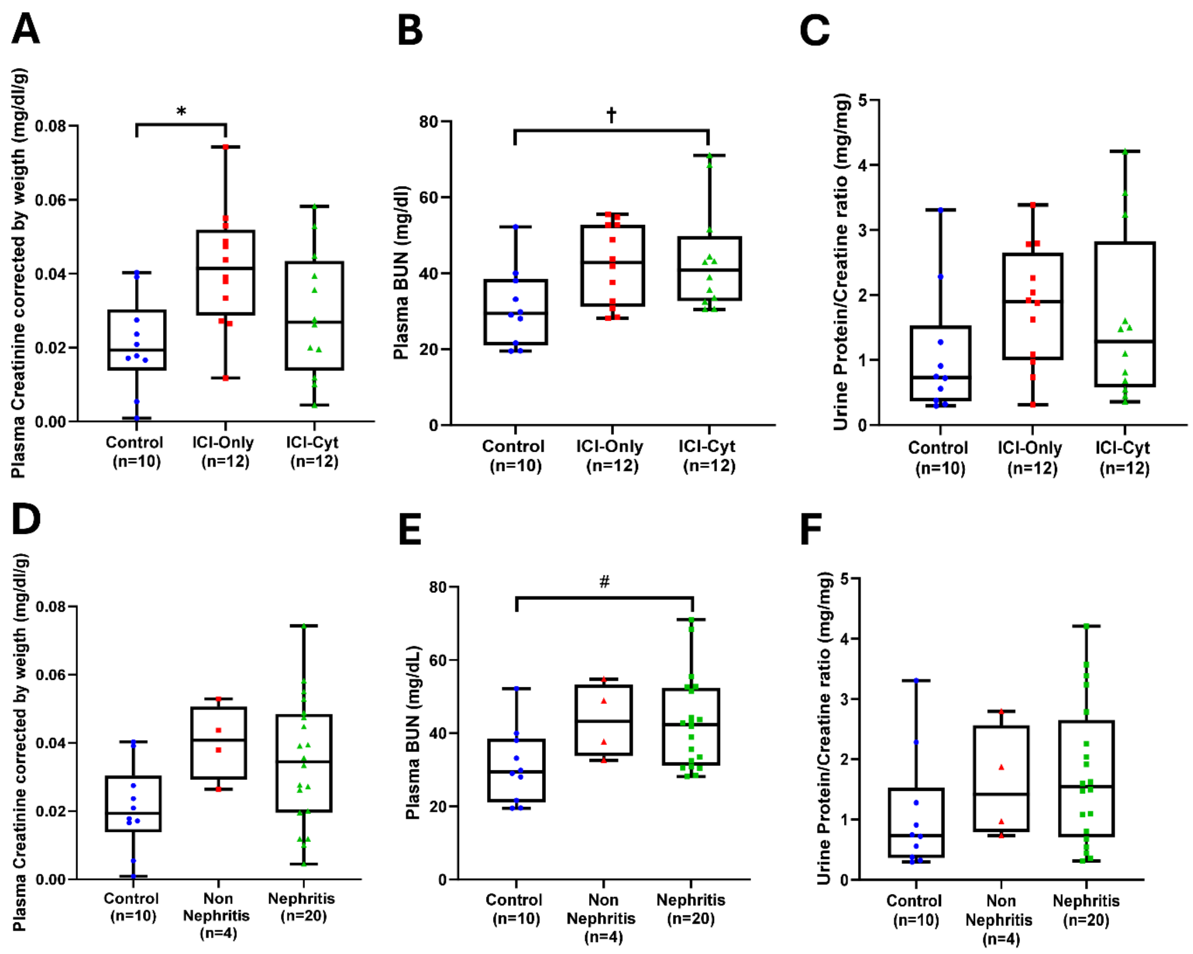

(A) Plasma creatinine corrected by body weight, **(B)** plasma blood urea nitrogen (BUN), and **(C)** urine protein-to-creatinine ratio (UPCR) across treatment groups. **(D-F)** Same analytes stratified by nephritis status.

* *p*< 0.05 for group ICI-Only vs Control in post-hoc test adjusted for multiple comparisons.

† *p*< 0.05 for group ICI-Cyt vs Control in post-hoc test adjusted for multiple comparisons.

# *p*< 0.05 for group Nephritis vs Control in post-hoc test adjusted for multiple comparisons.

Supplementary Fig S2. CD4+ and CD8+ Glomerular T Cell Number by Treatment

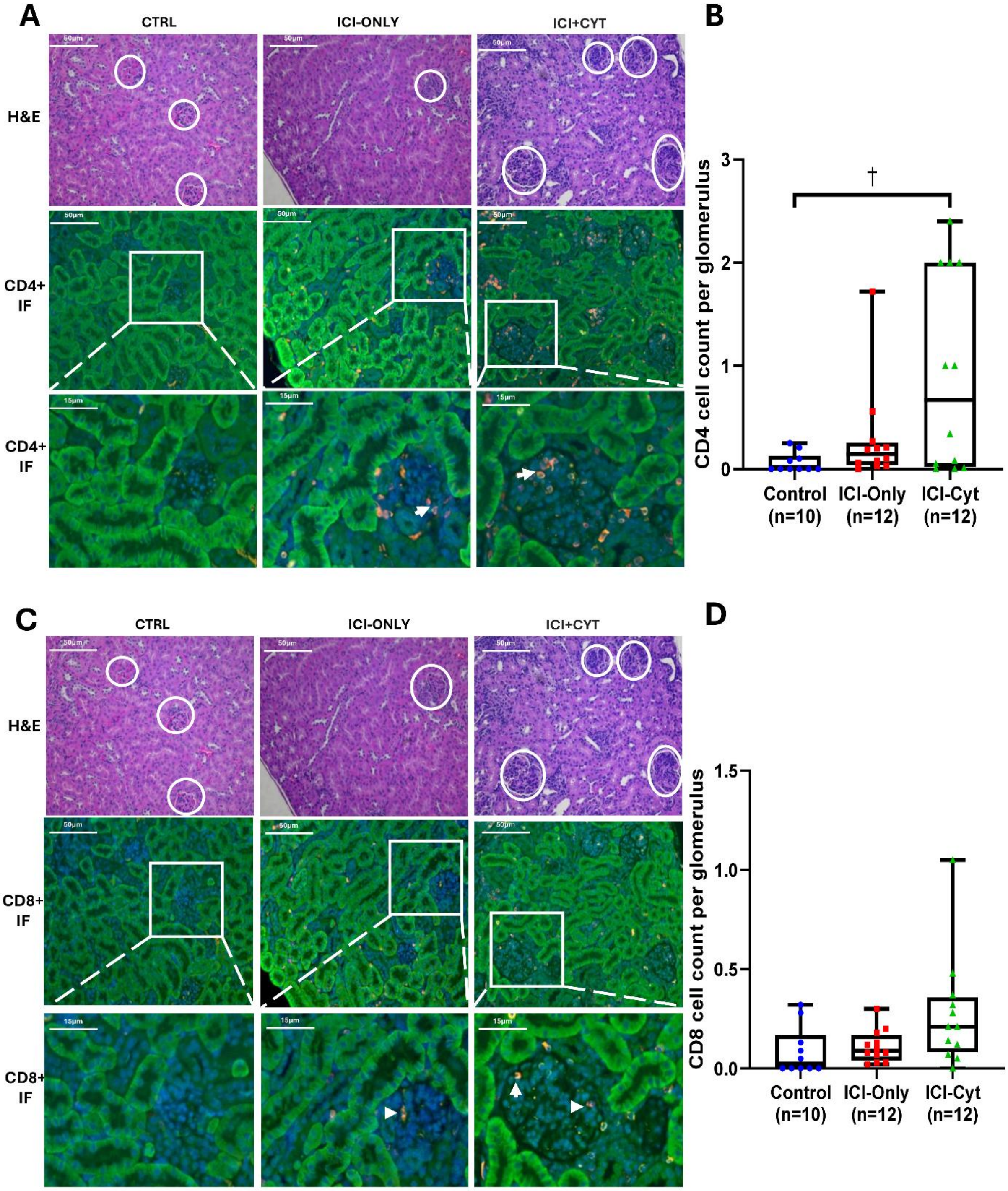

(A) Representative images of hematoxylin and eosin (H&E) staining (top row) and CD4+ immunofluorescence (IF) staining (middle and bottom rows). White circles highlight glomeruli in H&E and white rectangles plus arrowheads in IF. **(B)** Quantification of CD4+ per glomerulus across groups. **(C)** Representative images of H&E staining (top row) and CD8+ IF staining (middle and bottom rows). White circles highlight glomeruli in H&E and white rectangles plus arrowheads in IF. **(D)** Quantification of CD8+ per glomerulus across groups.
† $p<0.05$ for group ICI-Cyt vs Control in post-hoc test adjusted for multiple comparisons.

Supplementary Fig S3. CD4+ and CD8+ Glomerular and Tubular T Cell Number by Nephritis Status

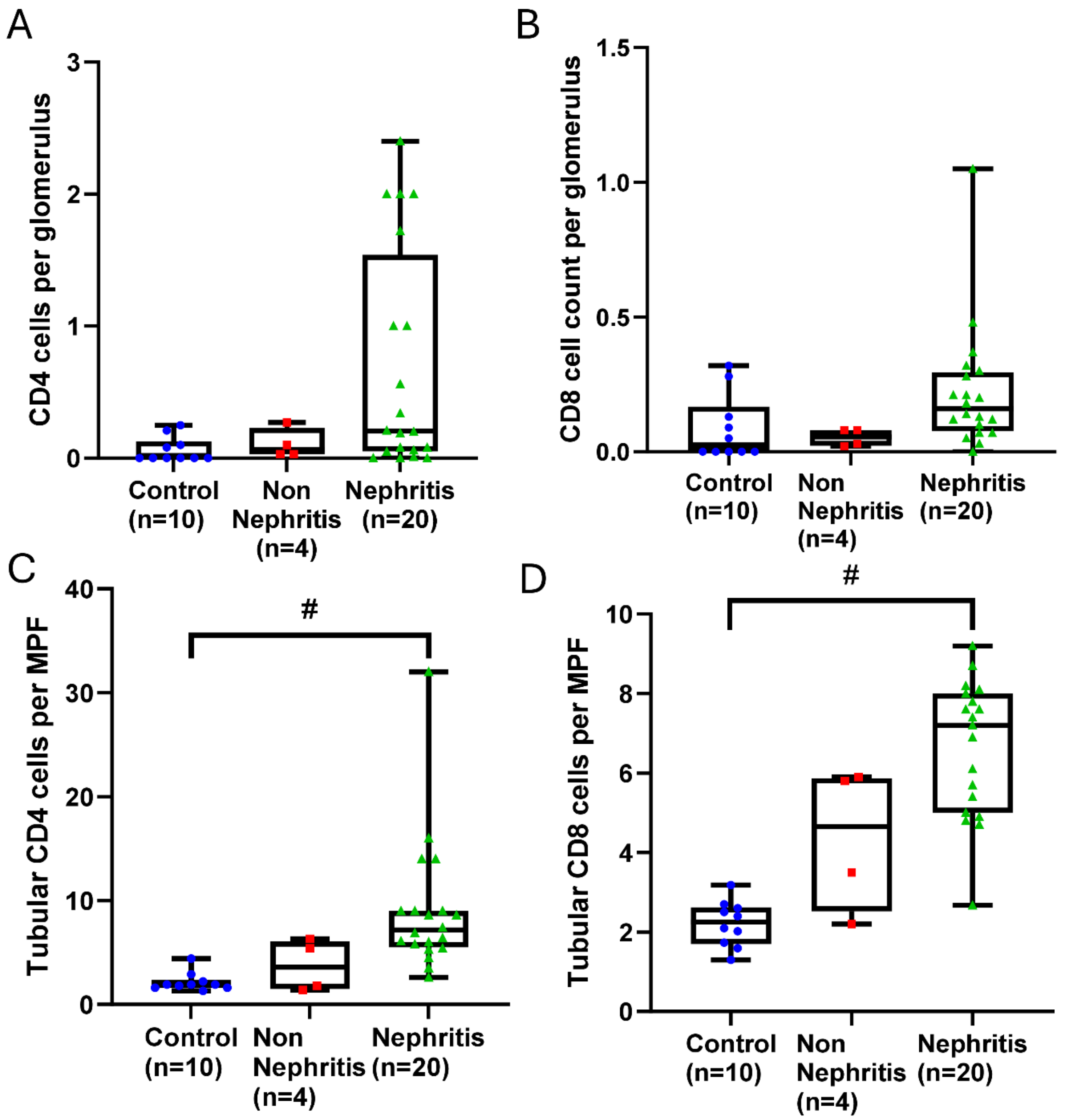

(A) CD4+ cell per glomerulus, **(B)** CD8+ cells per glomerulus, **(C)** tubular CD4+ cells per medium power field (MPF), and **(D)** tubular CD8+ cells per MPF across nephritis status group.

# *p*<0.05 for group Nephritis (median: 9 CD4+ cells, 7.2 CD8+ cells) vs Control group (median: 2.21 CD4 cells, 2.25 CD8+ cells) in post-hoc test adjusted for multiple comparisons.

Supplementary Fig. S4. Cytokine Concentration Profile in Kidney Tissue

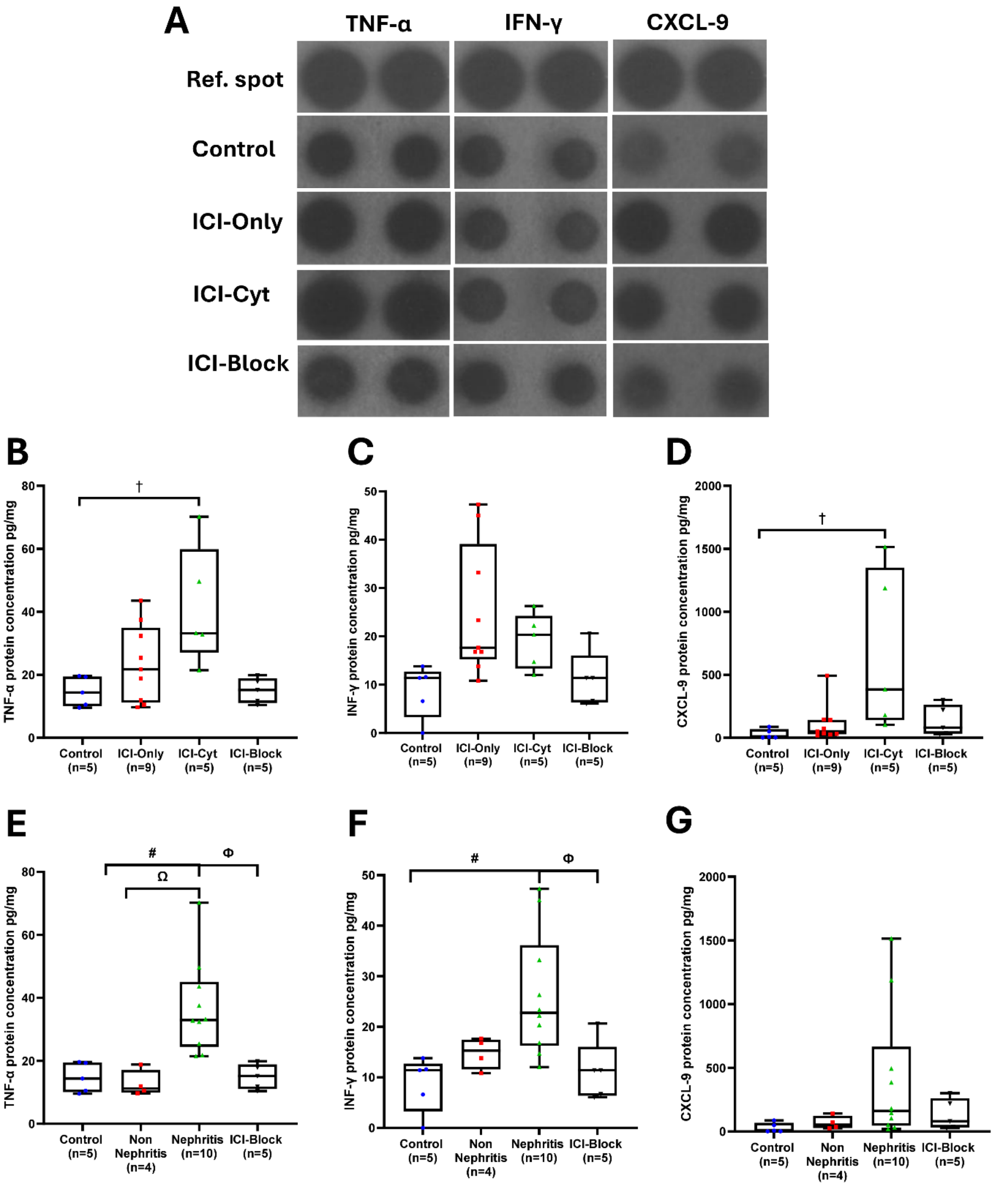

(A) TNF-α, IFN-γ, and CXCL-9 immunoblots from cytokine array by treatment group **(B-D)** TNF-α, IFN-γ, and CXCL-9 concentration by treatment groups. **(E-G)** TNF-α, IFN-γ, and CXCL-9 concentration by nephritis status groups.

† p<0.05 for group ICI-Cyt vs Control in post-hoc test adjusted for multiple comparisons.
# p<0.05 for group Nephritis vs Control in post-hoc test adjusted for multiple comparisons.
Φ p<0.05 for group Nephritis vs ICI-Block in post-hoc test adjusted for multiple comparisons.
Ω p<0.05 for group Nephritis vs Non-Nephritis in post-hoc test adjusted for multiple comparisons.

Supplementary Fig. S5. Kidney Function Parameters Across Treatment Groups and Nephritis Status Including TNF-α Blockade Group

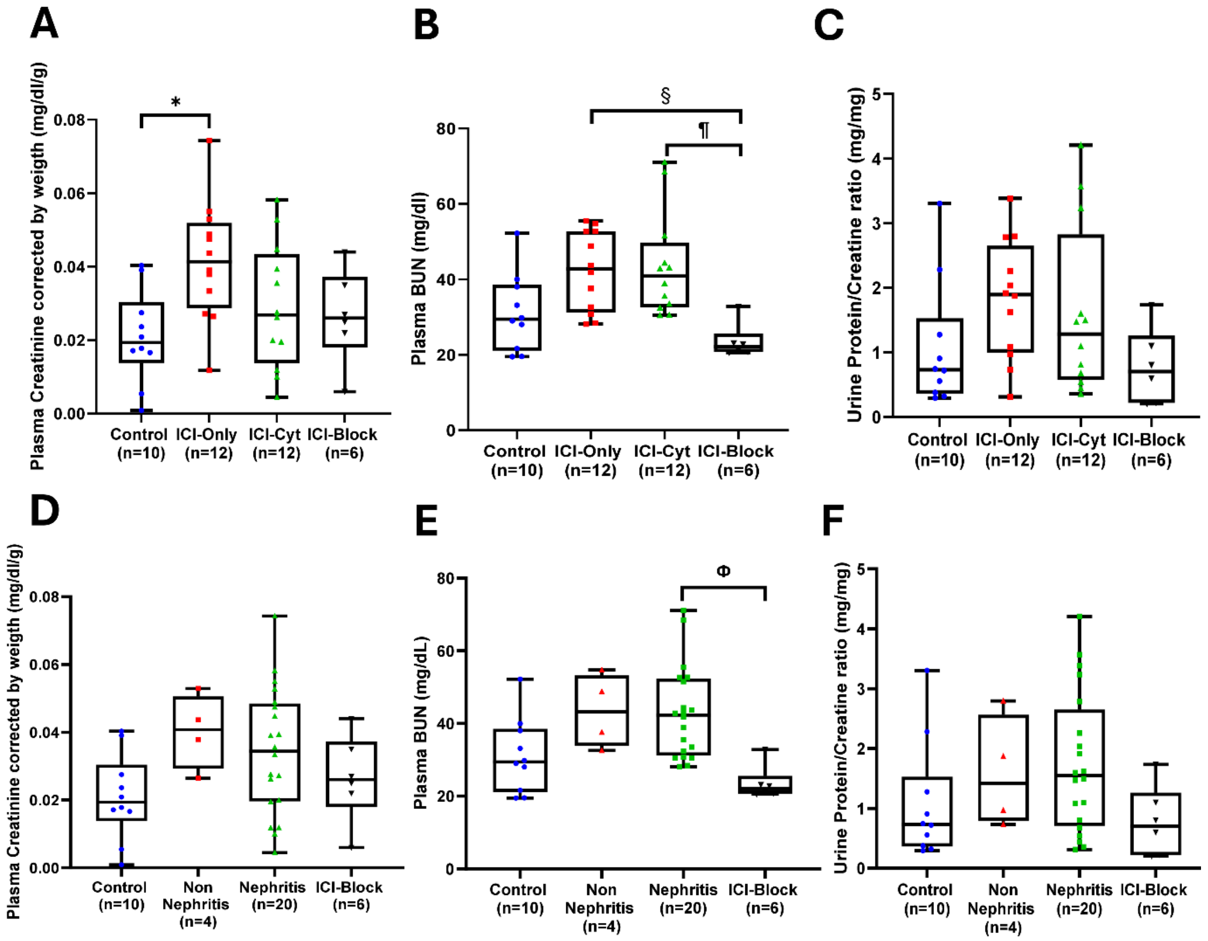

(A) Plasma creatinine corrected by body weight, **(B)** blood urea nitrogen (BUN**),** and **(C)** urine protein-to-creatinine ratio (UPCR) across treatment groups. **(D-F)** Same analytes stratified by nephritis status.

Treatment groups:

* *p*<0.05 for group ICI-Only vs Control in post-hoc test adjusted for multiple comparisons.

¶ *p*<0.05 for group ICI-Only vs ICI-Block in post-hoc test adjusted for multiple comparisons.

§ *p*<0.05 for group ICI-Cyt vs ICI-Block in post-hoc test adjusted for multiple comparisons.

Nephritis groups:

Φ *p*<0.05 for group Nephritis vs ICI-Block in post-hoc test adjusted for multiple comparisons.

Supplementary Fig. S6. Treatment-Dependent Changes in Kidney Cell Clusters Revealed by Single-Cell Transcriptomics

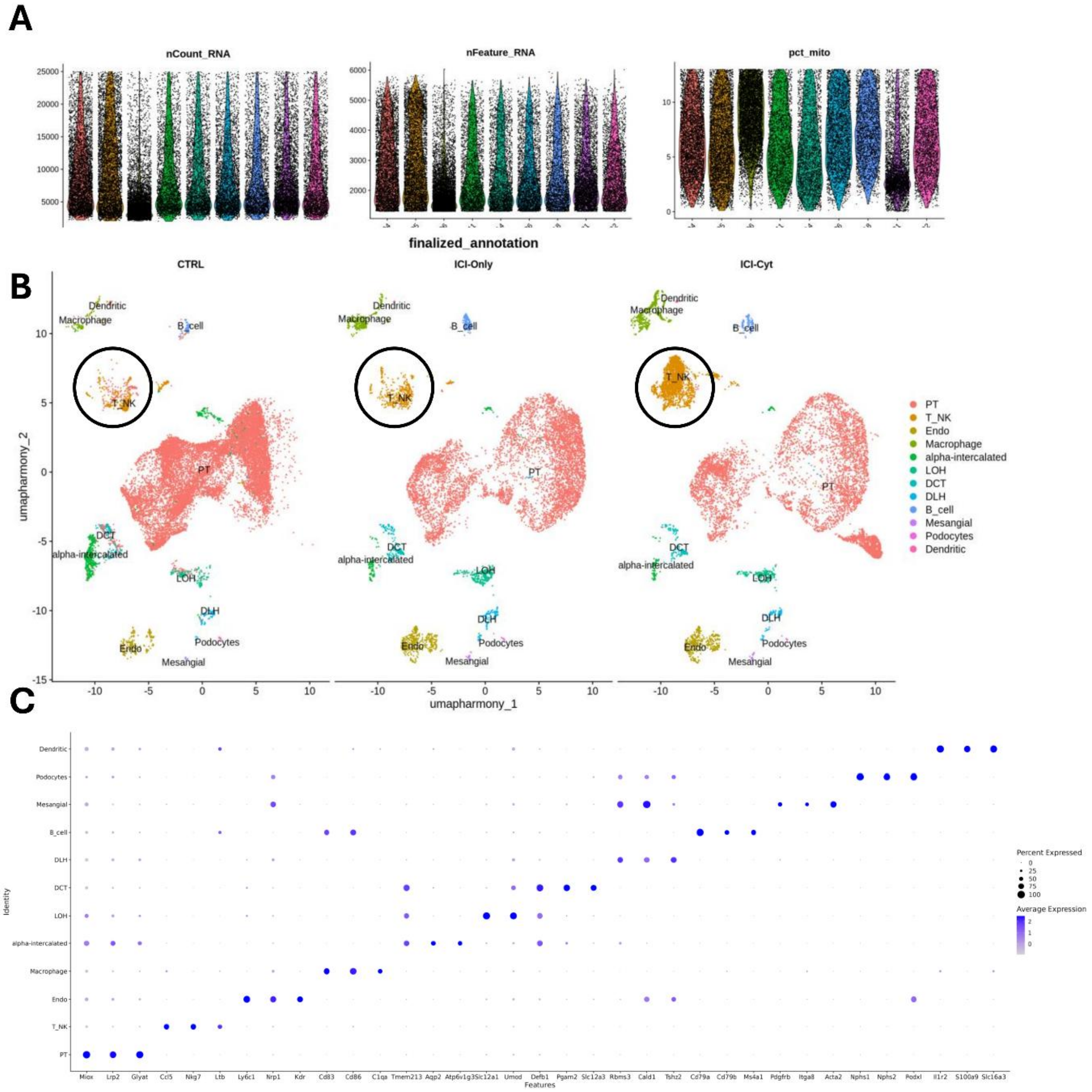

(A) Quality Control from samples **(B)** UMAP for different cell types. **(C)** Dot plot showing genes used to determine clusters.

Supplementary Fig. S7. Biological Analysis of Gene Expression for Downregulated Genes

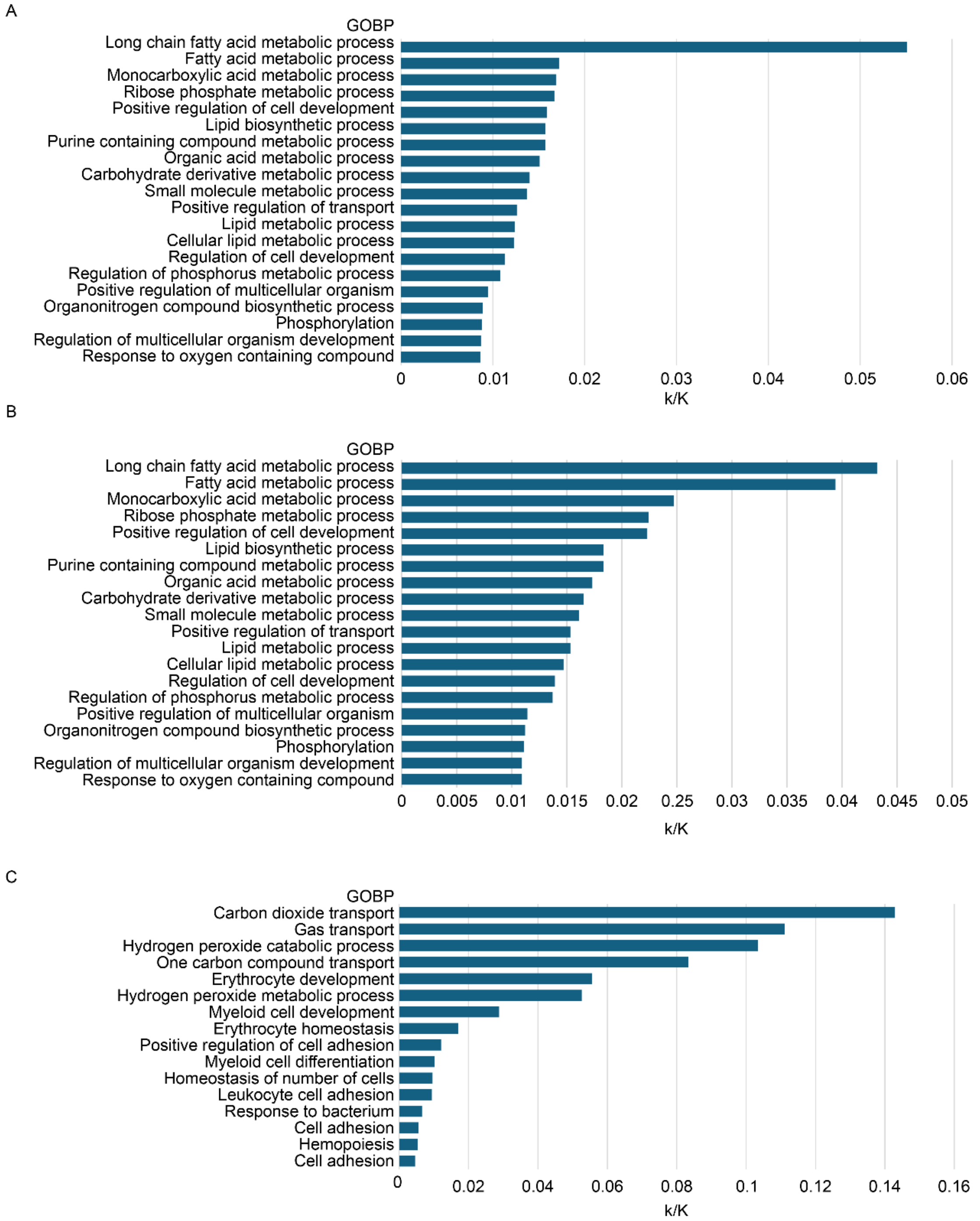

(A-B) Downregulated genes biological process analysis from gene set enrichment analysis (GSEA) for ICI-Cyt against Control CD4+ T cells and CD8+ effector cells, respectively. **(C)** Downregulated genes biological process analysis from GSEA for ICI-Cyt against ICI-Only CD4+ T cells.

References

1. Lee HW, Harris AN, Romero MF, et al. NBCe1-A is required for the renal ammonia and K+ response to hypokalemia. *Am J Physiol Renal Physiol*. 2020;318(2):F402-F421. doi:10.1152/ajprenal.00481.2019

2. International Organization for Standardization (ISO). ISO 10993-6:2016. Biological evaluation of medical devices—Part 6: Tests for local effects after implantation. Published online 2016. https://standards.iteh.ai/catalog/standards/sist/f011bf3e-641d-4762-b633-521285dd418e/iso-10993-6-2016

3. Chromium Next GEM Single Cell 3' Gene Expression v3.1 (Dual Index) User Guide - Rev F. Published online 2024. https://www.10xgenomics.com

4. Stewart BJ, Ferdinand JR, Young MD, et al. Spatiotemporal immune zonation of the human kidney. *Science*. 2019;365(6460):1461-1466. doi:10.1126/science.aat5031

5. Lake BB, Menon R, Winfree S, et al. An atlas of healthy and injured cell states and niches in the human kidney. *Nature*. 2023;619(7970):585-594. doi:10.1038/s41586-023-05769-3

6. Ianevski A, Giri AK, Aittokallio T. Fully-automated and ultra-fast cell-type identification using specific marker combinations from single-cell transcriptomic data. *Nat Commun*. 2022;13(1):1246. doi:10.1038/s41467-022-28803-w

7. Park J, Shrestha R, Qiu C, et al. Single-cell transcriptomics of the mouse kidney reveals potential cellular targets of kidney disease. *Science*. 2018;360(6390):758-763. doi:10.1126/science.aar2131

8. Balzer MS, Rohacs T, Susztak K. How Many Cell Types Are in the Kidney and What Do They Do? *Annu Rev Physiol*. 2022;84:507-531. doi:10.1146/annurev-physiol-052521-121841
